# Mechanism and Origins of Stereoselectivity of the Aldol-Tishchenko Reaction of Sulfinimines

**DOI:** 10.1021/acs.joc.0c02862

**Published:** 2021-02-15

**Authors:** Aneta Turlik, Kaori Ando, Pamela Mackey, Emma Alcock, Mark Light, Gerard P. McGlacken, K. N. Houk

**Affiliations:** † Department of Chemistry and Biochemistry, University of California, Los Angeles, California 90095-1569, United States; ‡ Department of Chemistry and Biomolecular Science, Faculty of Engineering, Gifu University, Yanagido 1-1, Gifu 501-1193, Japan; § School of Chemistry and Analytical and Biological Chemistry Research Facility, University College Cork, Cork, Ireland; ∥ School of Chemistry, University of Southampton, Southampton, SO17 1BJ, United Kingdom

## Abstract

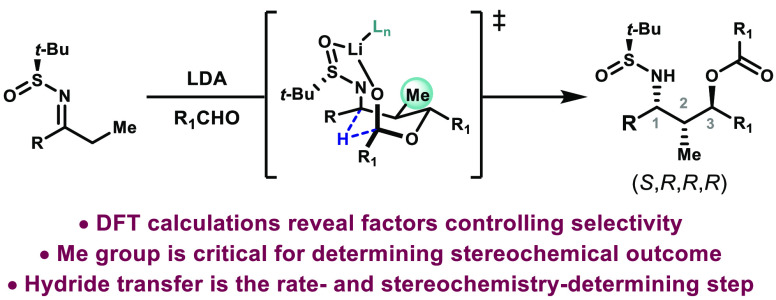

Density functional theory computations have elucidated the mechanism and origins of stereoselectivity in McGlacken’s aldol-Tishchenko reaction for the diastereoselective synthesis of 1,3-amino alcohols using Ellman’s *t*-butylsulfinimines as chiral auxiliaries. Variations of stereochemical outcome are dependent on the nature of the ketone starting materials used, and the aspects leading to these differences have been rationalized. The intramolecular hydride transfer step is the rate- and stereochemistry-determining step, and all prior steps are reversible.

The aldol-Tishchenko reaction is a powerful means for construction of 1,3-diols through the stereoselective reaction of an enolate with 2 equiv of aldehyde. While this type of reaction has been known for over 100 years,^1^ it was only in the 1990s that a mechanistic understanding of the transformation was developed.^2^ In subsequent years, the use of chiral ligands, such as BINOLs and cinchona alkaloids, made asymmetric variants possible.^3^

In 2015, McGlacken and co-workers reported the stereoselective synthesis of 1,3-amino alcohols through an aldol-Tishchenko process (Scheme 1).^4^ Diastereoselectivity was imparted through the use of *t*-butylsulfinimines, the Ellman auxiliaries.^5^ After an initial aldol reaction (**1** to **2**), the resulting alkoxide reacts with another equivalent of the aldehyde (**2** to **3**), followed by an intramolecular hydride transfer, to produce the sulfinamide (**4**). The ester and the chiral auxiliary can then be cleaved to produce a 1,3-amino alcohol.

**Scheme 1:**
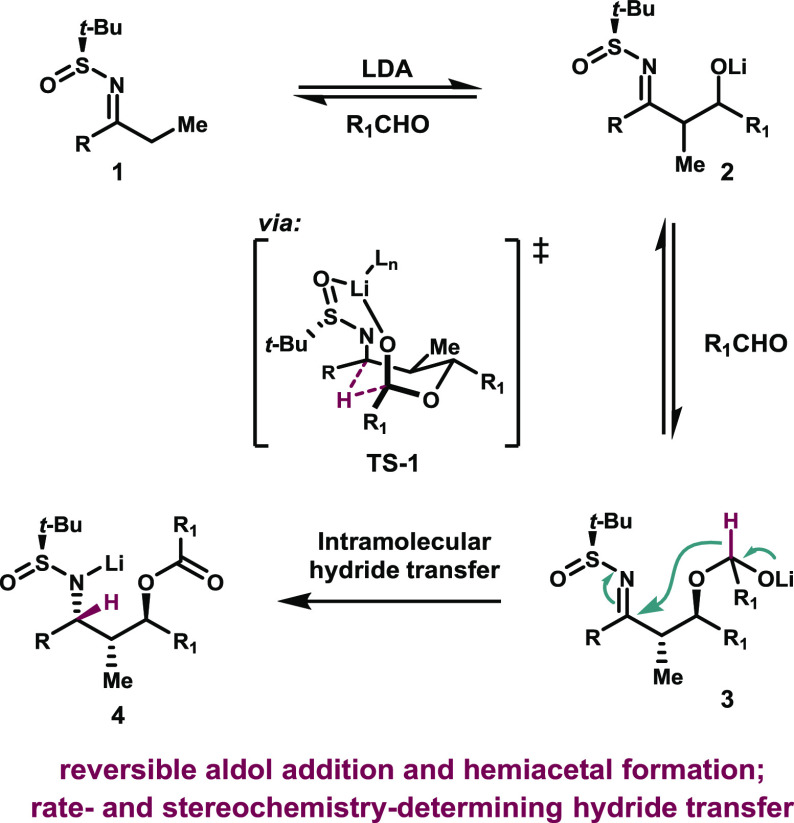
Synthesis of 1,3-Diols via the Aldol-Tishchenko Reaction

A variety of substrate classes could be used to form the aldol-Tishchenko product in high yield and diastereoselectivity, including sulfinimines derived from propiophenone, acetophenone, and dimethylcyclopentanone (Scheme 2). Surprisingly, the diastereochemical outcome of the reactions was different depending on the starting material: whereas all substrates produced the 1,3-*anti* product, the relative stereochemistry of the sulfinimine *t*-Bu group and the amine was *anti* in the case of the propiophenone-derived substrates, and *syn* in the case of the acetophenone- and dimethylcyclopentanone-derived sulfinimines.

**Scheme 2:**
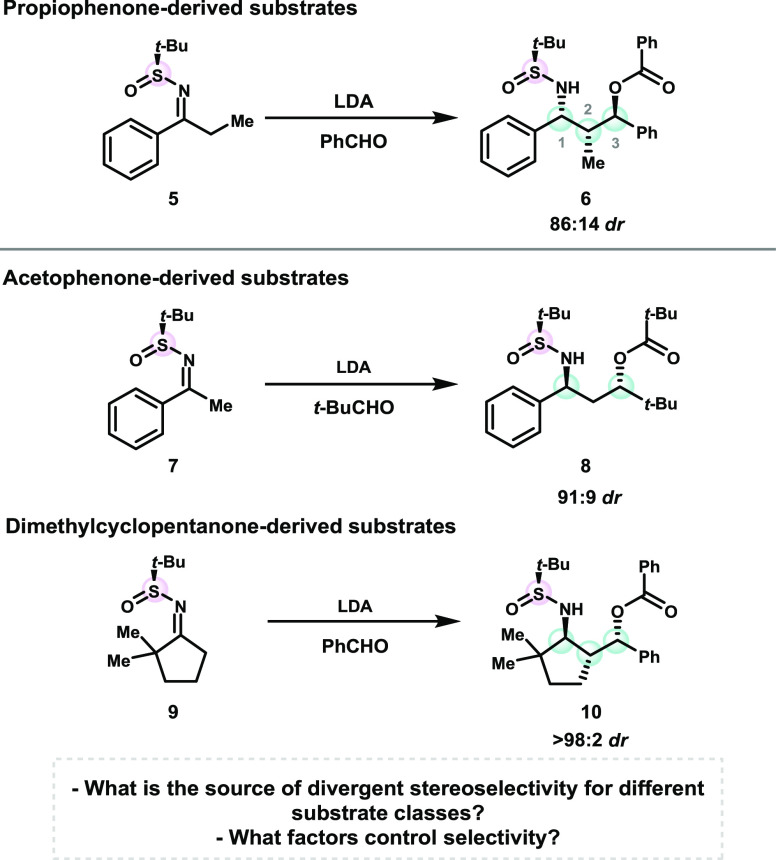
Divergent Stereochemical Results with Different Substrate Classes

These substrates differ both by the identity of the aldehyde that reacts with the sulfinimine, as well as by the substitution of the starting material. Experimentally, the use of pivaldehyde was not successful with the propiophenone-derived starting materials, and benzaldehyde was not successful with acetophenone-derived substrates, and thus these factors could not be easily disentangled through experimental studies.

We now report computational studies of the mechanism to determine what factors control the stereoselectivity of the reaction. In particular, we sought to determine why (1) the 1,3-*anti* product is favored in all cases and (2) the stereochemistry is different for different types of substrates. Furthermore, the stereochemical orientation of products from dimethylcyclopentanone-derived substrates has been unequivocally identified using crystallography. Despite the fact that deprotonation occurs at a methylene rather than a methyl group, the stereochemistry matches that of the acetophenone-derived substrates, not the propiophenone-derived ones. Again, this has been rationalized using extensive calculation.

DFT calculations were performed with Gaussian 16^6^ to analyze what factors control the stereoselectivity for different substrates. For each structure, an extensive conformer search was performed with CREST^7^ to locate the lowest-energy conformer of reactants and transition states. Geometry optimizations were performed with the B3LYP functional,^8^ augmented with Grimme’s D3 empirical dispersion term,^9^ and the 6-31G(d) basis set. Frequency calculations confirmed the optimized structures as minima (zero imaginary frequencies) or transition state structures (one imaginary frequency) on the potential energy surface. Intrinsic reaction coordinate (IRC) calculations were performed in order to connect the transition states to the reactants and the products. Single point energies were calculated using M06-2X-D3/6-311+G(d,p),^10^ and a quasi-harmonic correction was applied using the GoodVibes program.^11^

Experimental studies of the aldol-Tishchenko reaction by McGlacken and co-workers have shown that the steps leading to the final intramolecular reduction step are reversible, and that the reduction step determines the stereochemical outcome of the reaction.^4^ Thus, our calculations were focused on the reduction step in order to determine why a change in stereochemistry was observed between the propiophenone substrates compared to the acetophenone and dimethylcyclopentanone substrates, as well as what factors control selectivity. We first examined the propiophenone-derived sulfinimine, which formed product **6′** in 87% yield and 84:16 *dr* (Figure 1).^4^ In this case, one major diastereomer was observed out of a possible eight. The absolute stereochemistry of the major diastereomer was confirmed by X-ray crystallographic analysis as the (*S*,*R*,*R*,*R*)-diastereomer. The absolute configuration of the minor diastereomer has not been determined.

**Figure 1:**
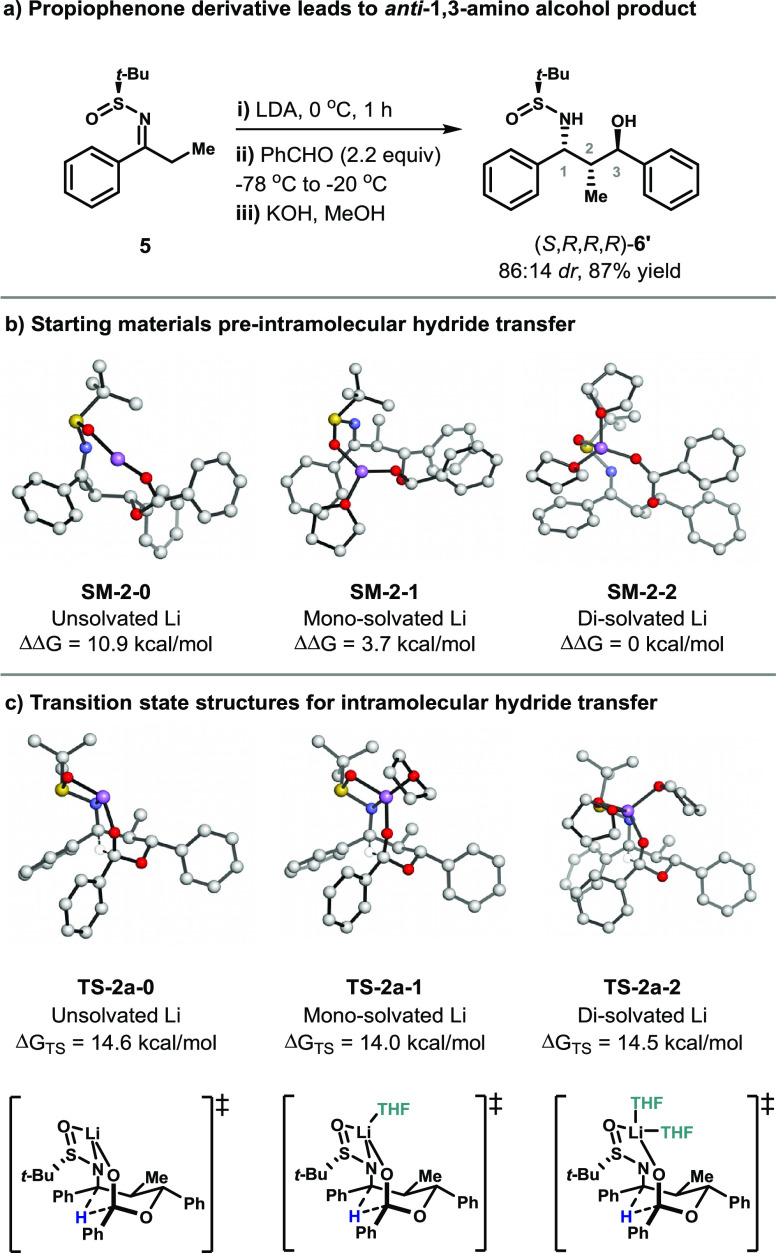
(a) Aldol-Tishchenko reaction of propiophenone-derived sulfinimine. (b) Free energies of starting material structures with various explicit solvation models. (c) Transition states for intramolecular hydride transfer reactions with various explicit solvation models.

Various solvation models were evaluated for computational modeling of the intramolecular hydride step of the reaction sequence. Inclusion of explicit solvents has been shown to be important for calculations of other Li complexes.^12^ The starting material for hydride transfer wherein Li is coordinated to two THF molecules was lowest in energy, compared to the mono-solvated (ΔΔ*G* = 3.7 kcal/mol) and unsolvated (ΔΔ*G* = 10.9 kcal/mol) systems. However, the activation barriers (Δ*G*_TS_) for the reduction step using the unsolvated, mono-solvated, and di-solvated complexes were similar: 14.6, 14.0, and 14.5 kcal/mol, respectively. In the mono-solvated complex, the lithium cation is tetra-coordinated and bound to one THF molecule, two *O*-atoms, and the *N*-atom. In the di-solvated case, the lithium cation loses *N*-coordination but remains tetra-coordinated, which lowers the energy of the complex. The di-solvated lithium complex was found to be the lowest in energy and was thus used for comparison of the factors affecting the hydride transition states for various substrates.

The formation of eight different diastereomers is possible in the aldol-Tishchenko reaction of propiophenone-derived starting material **5**. The transition state barriers for all eight diastereomers were calculated, and the three lowest-energy transition states are shown in Figure 2. Consistent with experimental results, formation of product (*S*,*R*,*R*,*R*)-**6** proceeds with the lowest-energy transition state barrier (14.5 kcal/mol). The difference between the two lowest transition state barriers (0.6 kcal/mol) is consistent with the experimental *dr* of 86:14 and would correspond to a *dr* of 84:16 at −78 °C and 78:22 at −20 °C.

**Figure 2:**
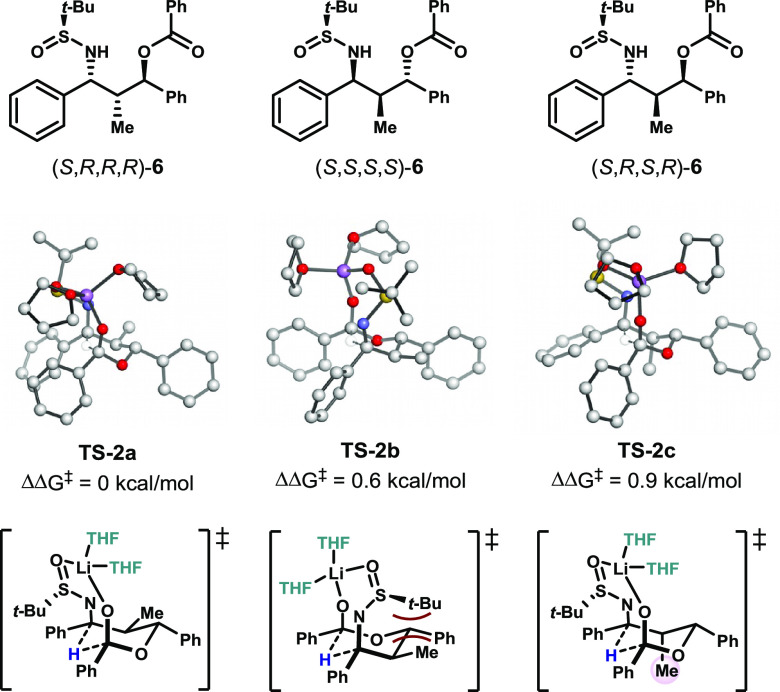
Transition state structures leading to the formation of three stereoisomers in the propiophenone series with the lowest-energy transition state barriers.

The reaction leading to major product (*S*,*R*,*R*,*R*)-**6** proceeds via a six-membered chair transition state (**TS-2a**) in which each of the substituents is placed in an equatorial orientation around the six-membered ring (Figure 2). In the lowest-energy transition state, the *t*-Bu group is optimally orientated away from the ring in order to avoid unfavorable steric interactions. This dictates the relationship between the S and C-N stereocenters. The relative relationship between the stereocenters at C1 and C3 is *trans* in the transition state for formation of the major product because this allows for the Ph group to be equatorial in the chairlike transition state. The transition states toward hydride transfer were also calculated for the second and third major contributors to the diastereomeric distribution. The second-lowest-energy transition state (**TS-2b**, ΔΔ*G*^⧧^ = 0.6 kcal/mol) leads to the stereochemistry analogous to that observed in the acetophenone-derived substrates. In this transition state structure, an unfavorable steric interaction exists between the equatorial methyl group and the *t*-Bu group of the sulfinimine, with a distance of 2.34 Å between these two groups. The penalty for placing the methyl substituent in an axial orientation is 0.9 kcal/mol, leading to **TS-2c**.

In the cases of the propiophenone substrate, the transition states containing the *Z*-isomer of the imine led to the major diastereomers of product. Computational results revealed that transition states containing the *E*-isomer were significantly higher in energy. For example, the transition state leading to the experimentally confirmed major diastereomer (*S*,*R*,*R*,*R*)-**6** using the *E*-isomer was found to be higher in energy by 6.4 kcal/mol in comparison to the *Z*-isomer (Figure 3). This may be due to the steric interactions between the sulfinimine *t*-Bu group and the C2 methyl group, which are only 2.28 Å apart. In addition, calculations predict that hydride transfer reactions in which only the *E*-isomer of the sulfinimine is accessible would lead predominantly to formation of a different diastereomer (*S*,*S*,*S*,*S*), which is inconsistent with experimental results. We thus conclude that the transition state for intramolecular hydride transfer in the propiophenone-derived series proceeds with the *Z*-isomer of the sulfinimine.

**Figure 3:**
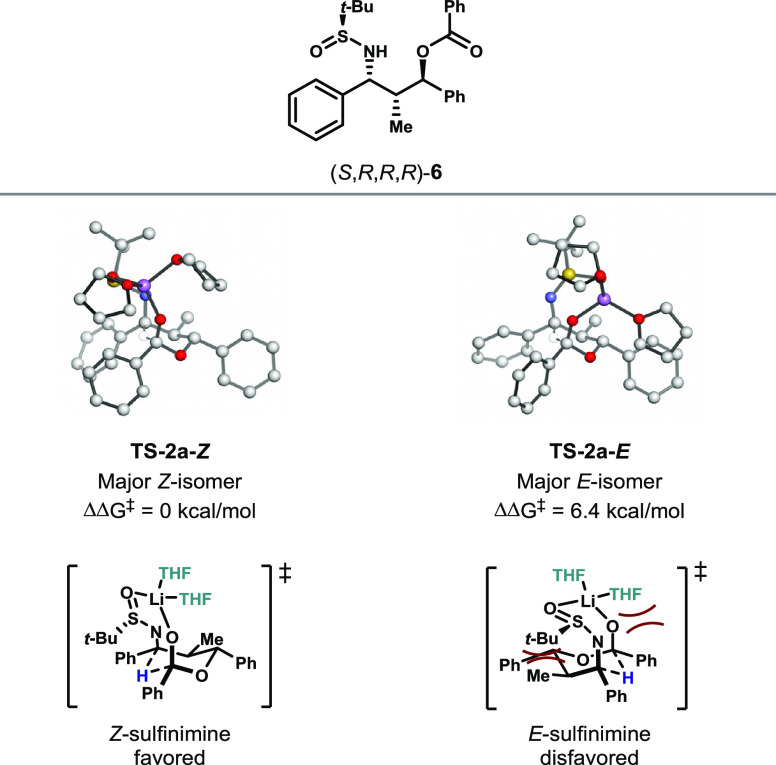
Comparison between *E*- and *Z*-isomers for the propiophenone series.

Experimentally, acetophenone substrate **7** formed product **8** in 70% yield and 91:9 *dr* (Figure 4).^6^ The absolute stereochemistry of the major diastereomer was assigned as (*S*,*S*,*S*) using X-ray crystallographic data.^6^ A reversal of absolute stereochemistry was observed for the acetophenone series (at C1 and C3) in comparison to the propiophenone series. In this system, there is a potential for the formation of four diastereomers, but only two diastereomers were observed under the reaction conditions. The absolute stereochemistry of the minor diastereomer was tentatively assigned (*S*,*R*,*R*) by comparison of all four diastereomerically pure samples, using ^1^H NMR and ^13^C NMR data.

**Figure 4:**
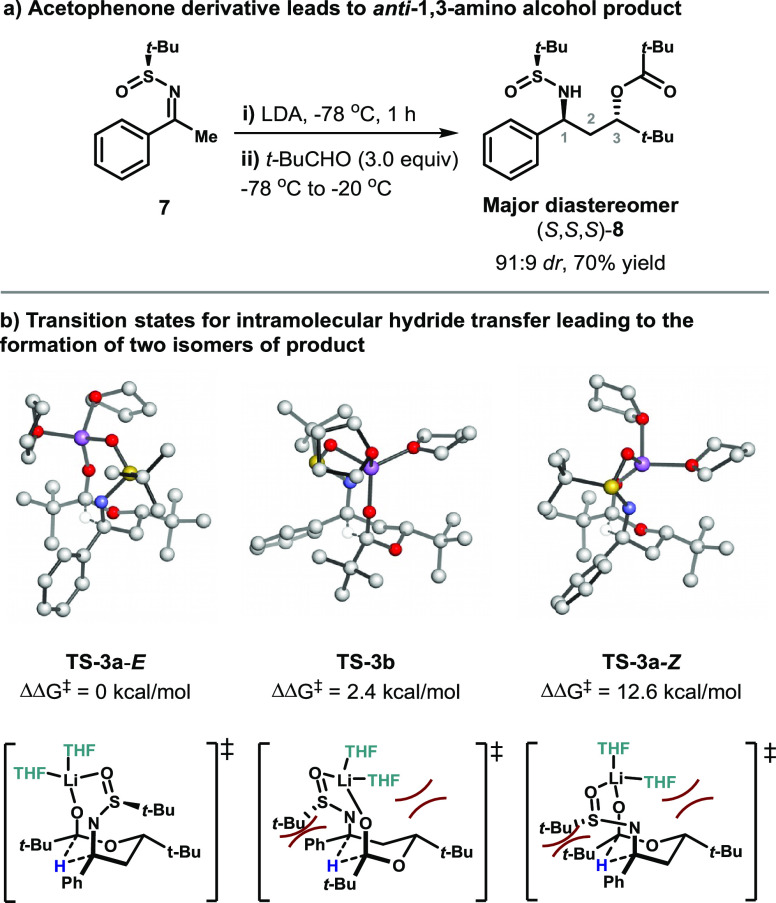
DFT calculations of transition states for hydride transfer in the acetophenone series.

A similar DFT analysis was performed for the acetophenone derived reaction (Figure 4). In this case, transition state **TS-3b** leading to formation of minor product (*S*,*R*,*R*)-**8** is higher in energy than transition state **TS-3a-*****E***, which leads to formation of the major product, by 2.4 kcal/mol. This calculated difference overestimates the diastereoselectivity of the reaction (91:9 *dr*), as it would correspond to a selectivity of over 99%, but is consistent with the correct stereochemical outcome. As depicted in Figure 4, unfavorable steric interactions between the large *t*-Bu groups appended to the six-membered ring and the THF molecules on Li in the minor transition state (**TS-3b**) lead to preference for formation of the major diastereomer through **TS-3a-*****E***, where these interactions are not present. In **TS-3b**, the distance between the *t-*Bu group at C3 and the THF molecule is 2.27 Å. The *t*-Bu group of the sulfinimine is also positioned closer to the Ph group attached to the imine. In the case of the acetophenone-derived substrates, the *E*-isomer is strongly preferred to the *Z*-isomer (by 12.6 kcal/mol) in order to avoid unfavorable steric interactions between the *t*-Bu group of the sulfinimine and the *t-*Bu group of the ester.

In order to test our hypothesis that the additional methyl substituent on the propiophenone substrate plays the main role in reversing the selectivity, and not the *t*-Bu vs Ph substituents on the aldehyde that participates in the aldol-Tishchenko reaction, we performed calculations using substrates lacking this methyl group (Figure 5). Indeed, when the methyl group was removed, the lowest-energy product was the one in which the relative stereochemistry of the sulfinimine *t*-Bu group and the amine was *syn*, as in the acetophenone-derived substrates, thus leading to a reversal of selectivity. The transition state barrier toward formation of the *syn* product was 2.1 kcal/mol lower than that toward formation of the *anti* product, which is close to the difference in transition state barriers for the two products formed from the acetophenone-derived substrates (**TS-3a-*****E*** vs **TS-3b**, ΔΔ*G*^⧧^ = 2.4 kcal/mol). Here, similar factors influence selectivity as in the acetophenone case (Figure 4). This suggests that substitution at C2 leads to the reversal of selectivity.

**Figure 5:**
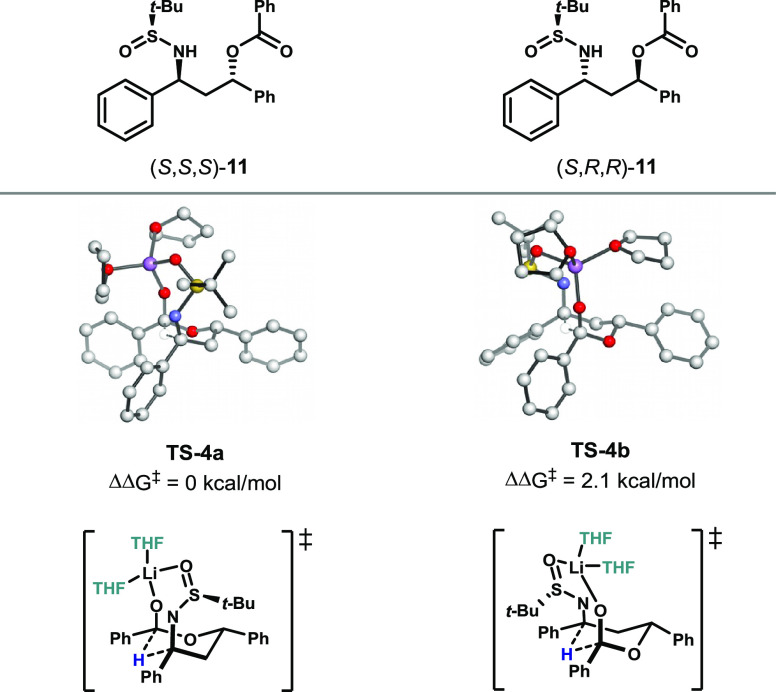
Comparison of des-methyl propiophenone series, and effects of *t*-Bu vs Ph substituents.

Analysis of these substrates computationally also allows for a direct comparison of the effects of the substituents derived from the aldehyde component (Ph in the reaction of **5** to **6** and *t*-Bu in the reaction of **7** to **8**). These model substrates in effect replace the Ph substituents in **TS-3** with *t*-Bu substituents. The stereochemical outcome is the same in the case of both *t*-Bu and Ph, with similar ΔΔ*G*^⧧^ for both (2.1 vs 2.4 kcal/mol). These results suggest that the steric and electronic differences between the *t*-Bu and Ph groups do not have a significant effect on stereoselectivity.

A similar analysis was performed with dimethylcyclopentyl substrate **9** (Figure 6). For this substrate, very good yields and excellent stereoselectivities were observed experimentally using pivaldehyde and benzaldehyde as aldol acceptors. The reaction of dimethylcyclopentyl sulfinimine **9** and benzaldehyde was chosen as our model. To investigate the origin of stereoselectivity for this reaction, eight transition states were located, three of which are shown in Figure 6. McGlacken and co-workers had tentatively assigned the stereochemistry by analogy to the propiophenone substrate, the (*S*,*R*,*R*,*R*)-**6′**, because a crystal structure could initially not be obtained.^4^ This stereochemistry would be obtained from a fully equatorial arrangement of the substituents during the six-membered ring transition state.

**Figure 6:**
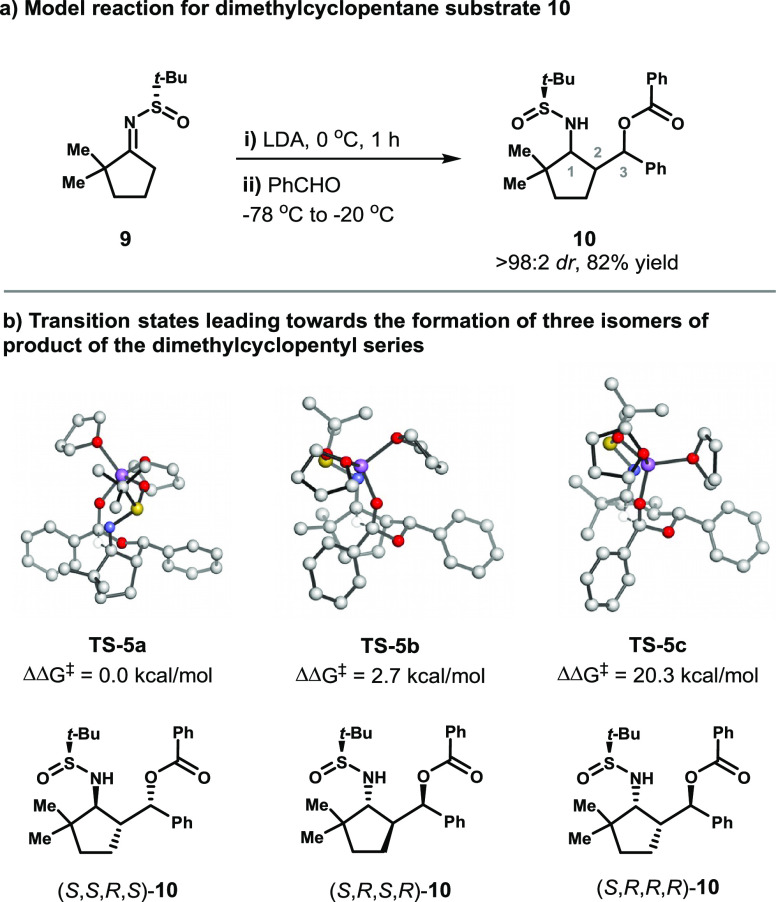
DFT studies of the transition states for hydride transfer in the dimethylcyclopentyl series.

A high-energy transition state barrier was obtained for the product with stereochemistry analogous to that of the propiophenone-derived product (**TS-5c**). This transition state was 20.3 kcal/mol higher in energy than the lowest-energy transition state **TS-5a** and led to product (*S*,*R*,*R*,*R*)-**10**. One of the lowest energy transition states, **TS-5b** is 2.7 kcal/mol higher in energy than the lowest-energy transition state, **TS-5a**. This energy difference corresponds to a *dr* of 99.9:0.1 at −78 °C and 99.5:0.5 at −20 °C and is thus consistent with the experimental *dr* of >98:2. Structure **TS-5b** differs from **TS-5c** by the stereochemistry of C2, which shows that the equatorial orientation of the cyclopentyl ring is highly disfavored.

The stereoselectivity of this reaction is in line with that of the acetophenone substrate, and similar steric interactions disfavor the minor diastereomer. Although an extra substituent is present at the C2 position, as in the propiophenone-derived substates, this substituent causes lesser steric strain when it is in the axial position. Conversely, the equatorial configuration of the cyclopentyl ring in **TS-5c** leads to highly unfavorable steric interactions between the *t*-Bu group of the sulfinimine and the *t*-Bu group on the cyclopentyl ring.

Further analysis of the NMR spectra suggests that the stereochemistry between positions C2 and C3 is *syn*, as evidenced by the low coupling constant between the protons at these positions. The high coupling constant between the protons at C1 and C2 suggests an *anti* relationship. Compound (*S*,*S*,*R*,*S*)-**10**, which is formed through **TS-5a**, satisfies both of these criteria.

The computationally predicted orientation of the dimethylcyclopentanone-derived products matched that of acetophenone rather than propiophenone-derived compounds, despite the fact that the nature of the deprotonation site is methylene rather than methyl. This somewhat surprising result merited a renewed attempt to crystallize some of the aldol-Tishchenko products from this series. Indeed, we obtained crystal structures of the products of the reactions of dimethylcyclopentyl imine **9** with 4-fluorobenzaldehyde and 4-methylbenzaldehyde. These crystal structures matched the computationally predicted stereochemistry, as in compound (*S*,*S*,*R*,*S*)-**10**. Similarly, the use of other benzaldehyde derivatives led to products with high diastereoselectivity (Table 1).

**Table 1:**
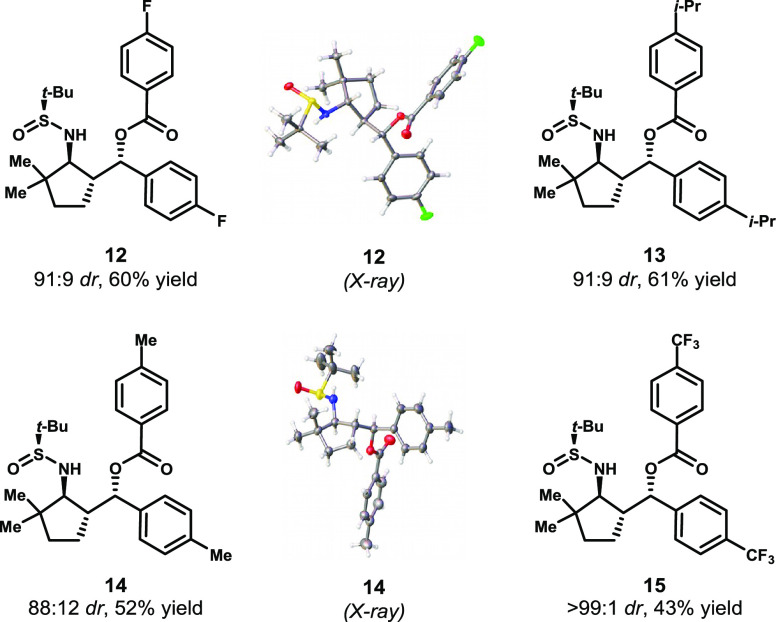
Products of Aldol-Tishchenko Reactions of Dimethylcyclopentyl Sulfinimine **9** with Aldehydes

The factors governing the selectivities of the aldol-Tishchenko reactions were determined by DFT calculations. Experimental investigations revealed an initial nonselective aldol step, and our computational studies focused on the irreversible and selectivity-determining intramolecular hydride transfer. These studies showed that the relationship between the C1 and C3 stereocenters is preferentially *anti* because this allows for the Ph or *t*-Bu group on C3 to be equatorial in the chairlike transition state. The cis/trans relationship between the S–*t*-Bu of the sulfinimine and C1–N is correlated to whether the transition state proceeds via the *Z*- or *E*-isomer of the sulfinimine: the *anti* product is formed through the *Z*-isomer, and the *syn* product is formed through the *E*-isomer. In the propiophenone-derived series, unfavorable steric interactions between the equatorial methyl group at C2 with the *t*-Bu group of the sulfinimine prevent the stereochemical outcome analogous to that observed with the acetophenone-derived substrates, and thus lead to a reversal of stereoselectivity.

Our study explains the origin of stereoselectivity for the aldol-Tishchenko reaction of sulfinylimines and provides a guide to the selectivities expected for related reactions. In addition, we demonstrate the power of the interplay between computational and synthetic chemistry for predictions of stereochemical outcome and structure determination.

## Experimental Section

### General Information

Solvents were dried and stored over flame-dried 4 Å molecular sieves (10–15% w/v) in a Young’s flask. The concentration of *n*-BuLi in hexanes was determined by titration with diphenylacetic acid. All aldehydes were freshly distilled prior to use and stored under an inert atmosphere. All other reagents were purchased from Sigma-Aldrich, Fluorochem, Alfa Aesar, and Acros unless otherwise noted. All nonaqueous reactions were carried out under an oxygen-free nitrogen atmosphere using oven-dried glassware.

Wet flash column chromatography was carried out using Kieselgel silica gel 60, 0.040–0.063 mm (Merck). Thin layer chromatography (TLC) was carried out on precoated silica gel plates (Merck 60 PF254). Visualization was achieved by UV and potassium permanganate staining. Melting points were measured on a Thomas Hoover Capillary Melting Point apparatus. Infrared (IR) spectra were recorded on a PerkinElmer FT-IR Paragon 1000 spectrophotometer.

NMR spectra were run in CDCl_3_ using TMS as the internal standard at 25 °C. ^1^H NMR (600 MHz) spectra, ^1^H NMR (400 MHz) spectra, and ^1^H NMR (300 MHz) spectra were recorded on Bruker Avance 600, Bruker Avance 400, and Bruker Avance 300 NMR spectrometers, respectively. ^13^C (150.9 MHz) spectra, ^13^C (100.6 MHz) spectra, and ^13^C (75.5 MHz) spectra were recorded on Bruker Avance 600, Bruker Avance 400, and Bruker Avance 300 NMR spectrometers, respectively, in proton decoupled mode. All spectra were recorded at University College Cork. Chemical shifts δ_H_ and δ_C_ are expressed as parts per million (ppm), positive shift being downfield from TMS; coupling constants (*J*) are expressed in hertz (Hz). Splitting patterns in ^1^H NMR spectra are designated as s (singlet), *br* s (broad singlet), d (doublet), dd (doublet of doublets), dt (doublet of triplets), t (triplet), q (quartet), quin (quintet), sext (sextet), sept (septet), and m (multiplet). For ^13^C NMR spectra, the number of attached protons for each signal was determined using the DEPT pulse sequence run in the DEPT-90 and DEPT-135 modes. COSY, HSQC, and HMBC experiments were performed to aid the NMR assignment of novel chemical structures.

Low-resolution mass spectra were recorded on a Waters Quattro Micro triple quadrupole instrument in electrospray ionization (ESI) mode using 50% acetonitrile-water, containing 0.1% formic acid as the mobile phase. Samples were made up in acetonitrile at a concentration of *ca*. 1 mg/mL. High-resolution mass spectra were recorded on a Waters LCT Premier TOF LC-MS instrument in electrospray ionization (ESI) mode using 50% acetonitrile-water, containing 0.1% formic acid as the mobile phase. Samples were made up in acetonitrile at a concentration of *ca*. 1 mg/mL.

Optical rotations were recorded on a DigiPol 781 TDV Polarimeter at 589 nm or on an Autopol V Plus Automatic Polarimeter at 589 nm in a 10 cm cell. Concentrations (*c*) are expressed in g/100 mL; [α]_D_^*T*^ is the specific rotation of a compound and is expressed in units of 10^–1^ deg cm^2^ g^–1^. The specific rotations were recorded to indicate the direction of enantioselection, and optically active samples are numbered with either (+)- or (−)- as prefix.

Single crystal X-ray data were collected at the University of Southampton using a Rigaku AFC12 FRE-HF diffractometer equipped with an Oxford Cryosystems low-temperature device, operating at *T* = 100 K. The structure was solved with the ShelXT (Sheldrick, 2015) structure solution program using the Intrinsic Phasing solution method and by using Olex2 as the graphical interface. The model was refined with version 2016/6 of ShelXL (Sheldrick, 2015) using Least Squares minimization. Most hydrogen atom positions were calculated geometrically and refined using the riding model, but some hydrogen atoms were refined freely.

^1^H NMR spectra, ^13^C NMR spectra, and LRMS and IR analyses were recorded for all previously prepared compounds. For novel compounds, in addition to the previously mentioned analysis, HRMS was also obtained.

In most cases, it was possible to separate the diastereomers; however, the yield reflects the mixture of diastereomers. The major diastereomer was fully characterized in all cases. Diastereoselectivity was determined by analysis of the ^1^H NMR spectrum of the crude reaction mixture.

### Synthesis of Starting Materials

#### General Procedure for the Synthesis of (*S*)-*tert-*Butyl Sulfinimine

To a solution of titanium ethoxide (2.0 equiv) in THF (4 mL per mmol of ketone) were added ketone (1.0 equiv) and (*S*)-*tert*-butanesulfinamide (1.0 equiv). The resulting mixture was heated at reflux using an oil bath. The reaction progress was monitored by TLC analysis. Once the reaction had gone to completion, brine (4 mL per mmol of ketone) was added and allowed to stir vigorously for 30 min. The slurry was then filtered through a pad of Celite and thoroughly washed with Et_2_O. The organic layer was dried over anhydrous MgSO_4_, filtered, and concentrated under reduced pressure to afford the crude (*S*)-*tert*-butyl sulfinimine which was purified using column chromatography on silica gel.

##### (*S*,*E*)-2-Methyl-*N*-(2,2-dimethylcyclopentylidene)propane-2-sulfinamide, **9**

Compound **9** was prepared using 2,2-dimethylcyclopentanone (1.12 mL, 8.9 mmol) and (*S*)-*tert*-butanesulfinamide (1.08 g, 8.9 mmol) according to the previously reported procedure.^4^ The crude compound was purified using column chromatography on silica gel (3:1, hexane:EtOAc) to give the title compound **9** as a pale yellow oil (1.19 g, 62%). Spectroscopic characteristics were consistent with previously reported data.^4^ [α]_D_^25^ +217.20 (*c* 0.5, CHCl_3_) (lit.^4^ [α]_D_^23^ +239.0 (*c* 1.0, CHCl_3_)). IR *v*_max_ (NaCl): 1636 (C=N stretch), 1089 (S=O stretch) cm^–1^. ^1^H NMR (300 MHz, CDCl_3_) δ 3.07–2.95 (1H, ddd, *J* = 19.3, 8.4, 1.1 Hz), 2.69–2.57 (1H, ddd, *J* = 19.4, 8.0, 1.6 Hz), 1.69–1.60 (2H, m), 1.91–1.76 (2H, m), 1.24 (9H, s), 1.12, 1.11 (2 × 3H, s) ppm. ^13^C NMR{^1^H} (75.5 MHz, CDCl_3_) δ 198.2, 56.7, 46.8, 38.7, 32.9, 26.3, 26.0, 22.3, 21.4 ppm. MS (ESI) *m*/*z*: 216 (M + H)^+^.

#### Synthesis of Products

To a Schlenk tube under a N_2_ atmosphere, containing diisopropylamine (1.2 equiv) in anhydrous THF (5 mL), was added *n*-BuLi (1.1 equiv) at 0 °C. The mixture was allowed to stir at 0 °C for 20 min to generate a solution of LDA. *tert*-Butanesulfinimine **9** (1.0 equiv) was then added slowly (neat), dropwise at 0 °C. After the reaction mixture was allowed to stir for 1 h at 0 °C, the solution was cooled to −78 °C and freshly distilled aldehyde (3.0 equiv) was added slowly (neat), dropwise. The reaction mixture was kept at −78 °C for 3 h and was allowed warm to −20 °C over 16 h.

#### Work-Up Conditions as per 1 mmol of Sulfinimine

The reaction was quenched with sat. aq. NH_4_Cl solution (1.5 mL). Sat. aq. NH_4_Cl (10 mL) was added and the mixture was extracted with EtOAc (3 × 20 mL). The organic layers were combined, dried over anhydrous MgSO_4_, filtered, and concentrated under reduced pressure to afford the crude product which was purified using column chromatography on silica gel.

##### (*S*)-((1*R*,2*S*)-2-(((S*)*-*tert*-Butylsulfinyl)amino)-3,3-dimethylcyclopentyl)(4-fluorophenyl)methyl 4-Fluorobenzoate, **12**

Compound **12** was prepared using sulfinimine **9** (0.215 g, 1 mmol) and 4-fluorobenzaldehyde (0.32 mL, 3 mmol). The crude compound (91:9 *dr*) was purified using column chromatography on silica gel (1:1, hexane:EtOAc) to give the title compound **12** as a white solid (0.278 g, 60% (mixture of diastereomers)). Major diastereomer: Mp 157–159 °C. [α]_D_^25^ +25.6 (*c* 0.5, CHCl_3_). IR *v*_max_ (NaCl): 3433 (N-H stretch), 1638 (C=O stretch), 1110 (C-N stretch) cm^–1^. ^1^H NMR (300 MHz, CDCl_3_) δ 8.12–8.07 (2H, m), 7.30–7.26 (2H, m), 7.19–7.13 (2H, m),7.03–6.98 (2H, m), 6.18 (1H, d, *J* = 2.4 Hz), 3.34 (1H, d, *J* = 9.8 Hz), 3.12 (1H, t, *J* = 9.8 Hz), 2.33–2.19 (1H, m), 1.97–1.87 (1H, m), 1.67–1.52 (3H, m), 1.27 (9H, s), 1.17, 0.92 (2 × 3H, s) ppm. ^13^C NMR{^1^H} (75.5 MHz, CDCl_3_) δ 166.5 (d, ^1^*J*_C-F_ = 254.8 Hz), 164.5, 162.5 (d, ^1^*J*_C-F_ = 246.5 Hz), 136.2 (d, ^4^*J*_C-F_ = 3.3 Hz), 132.1 (d, ^3^*J*_C-F_ = 9.3 Hz), 127.3 (d, ^3^*J*_C-F_ = 8.2 Hz), 126.3 (d, ^4^*J*_C-F_ = 3.2 Hz), 115.8 (d, ^2^*J*_C-F_ = 22.0 Hz), 115.4 (d, ^2^*J*_C-F_ = 21.4 Hz), 74.2, 69.0, 56.3, 51.7, 40.6, 38.6, 27.3, 22.9, 22.0, 20.2 ppm. HRMS (ESI) *m*/*z* calcd for C_25_H_32_F_2_NO_3_S [M + H]^+^: 464.2065, found 464.2065.

##### (*S*)-((1*R*,2*S*)-2-(((*S*)-*tert*-Butylsulfinyl)amino)-3,3-dimethylcyclopentyl)(*p*-tolyl)methyl 4-Methylbenzoate, **13**

Compound **13** was prepared using sulfinimine **9** (0.215 g, 1 mmol) and 4-methylbenzaldehyde (0.35 mL, 3 mmol). The crude compound (88:12 *dr*) was purified using column chromatography on silica gel (3:1, hexane:EtOAc) to give the title compound **13** as a white solid (0.236 g, 52% (mixture of diastereomers)). Major diastereomer: Mp 140–142 °C. [α]_D_^25^ +17.8 (*c* 0.5, CHCl_3_). IR *v*_max_ (NaCl): 3425 (N-H stretch), 1641 (C=O stretch), 1104 (C-N stretch) cm^–1^. ^1^H NMR (300 MHz, CDCl_3_) δ 7.99–7.96 (2H, m), 7.29–7.26 (2H, m), 7.20–7.18 (2H, m), 7.12–7.09 (2H, m), 6.15 (1H, d, *J* = 2.2 Hz), 3.28 (1H, d, *J* = 9.8 Hz), 3.15 (1H, t, *J* = 9.8 Hz), 2.30, 2.43 (2 × 3H, s), 2.30–2.19 (1H, m), 2.04–1.94 (1H, m), 1.73–1.53 (3H, m), 1.27 (9H, s), 1.17, 0.91 (2 × 3H, s) ppm. ^13^C NMR{^1^H} (75.5 MHz, CDCl_3_) δ 165.6, 143.8, 137.8, 137.1, 129.6, 129.2, 129.1, 127.6, 125.5, 74.3, 69.2, 56.3, 51.9, 40.6, 38.7, 27.2, 22.9, 21.9, 21.1, 21.7, 20.2 ppm. HRMS (ESI) *m*/*z* calcd for C_27_H_38_NO_3_S [M + H]^+^: 456.2567, found 456.2565.

##### (*S*)-((1*R*,2*S*)-2-(((*S*)-*tert*-Butylsulfinyl)amino)-3,3-dimethylcyclopentyl)(4-isopropylphenyl)methyl 4-Isopropylbenzoate, **14**

Compound **14** was prepared using sulfinimine **9** (0.215 g, 1 mmol) and 4-isopropylbenzaldehyde (0.45 mL, 3 mmol). The crude compound (91:9 *dr*) was purified using column chromatography on silica gel (3:1, hexane:EtOAc) to give the title compound **14** as a white solid (0.312 g, 61% (mixture of diastereomers)). Major diastereomer: Mp 105–107 °C. [α]_D_^25^ +14.25 (*c* 0.5, CHCl_3_). IR *v*_max_ (NaCl): 3426 (N-H stretch), 1640 (C=O stretch), 1107 (C-N stretch), 1054 (S=O stretch) cm^–1^. ^1^H NMR (300 MHz, CDCl_3_) δ 8.04–8.00 (2H, m), 7.35–7.32 (2H, m), 7.26–7.14 (4H, m), 6.17 (1H, d, *J* = 2.4 Hz), 3.36 (1H, d, *J* = 10.1 Hz), 3.15 (1H, t, *J* = 10.1 Hz), 2.98, 2.86 (2 × 1H, sept, *J* = 7.0 Hz), 2.30–2.19 (1H, m), 2.04–1.89 (1H, m), 1.73–1.52 (3H, m), 1.30–1.27 (9H, s, H-10 and 2 × 3H, d, *J* = 6.9 Hz), 1.18, 0.91 (2 × 3H, s) ppm. ^13^C NMR{^1^H} (75.5 MHz, CDCl_3_) δ 165.6, 154.6, 148.0, 138.0, 129.8, 128.0, 126.7, 126.5, 125.6, 74.3, 69.4, 56.4, 51.8, 40.6, 38.7, 33.7, 34.3, 27.2, 23.7, 23.9, 22.9, 21.8, 20.3 ppm. HRMS (ESI) *m*/*z* calcd for C_31_H_46_NO_3_S [M + H]^+^: 512.3193, found 512.3196.

##### (*S*)-((1*R*,2*S*)-2-(((*S*)-*tert*-Butylsulfinyl)amino)-3,3-dimethylcyclopentyl)(4-(trifluoromethyl)phenyl)methyl 4-(Trifluoromethyl)benzoate, **15**

Compound **15** was prepared using sulfinimine **9** (0.215 g, 1 mmol) and 4-isopropylbenzaldehyde (0.45 mL, 3 mmol). The crude compound (>99:1 *dr*) was purified using column chromatography on silica gel (4:1, hexane:EtOAc) to give the title compound **15** as a sticky colorless oil (0.242 g, 43% (mixture of diastereomers)). Major diastereomer: [α]_D_^25^ +26.4 (*c* 0.5, CHCl_3_). IR *v*_max_ (NaCl): 3292 (N-H stretch), 1730 (C=O stretch), 1128 (C-N stretch), 1067 (S=O stretch) cm^–1^. ^1^H NMR (600 MHz, CDCl_3_) δ 8.20 (2H, d, *J* = 8.1 Hz), 7.78 (2H, d, *J* = 8.1 Hz), 7.58 (2H, d, *J* = 8.1 Hz), 7.42 (2H, d, *J* = 8.1 Hz), 6.27 (1H, d, *J* = 2.1 Hz), 3.42 (1H, d, *J* = 9.5 Hz), 3.15 (1H, t, *J* = 9.9 Hz), 2.36–2.28 (1H, m), 1.99–1.83 (1H, m), 1.66–1.53 (3H, m), 1.28 (9H, s), 1.18, 0.93 (2 × 3H, s) ppm. ^13^C NMR{^1^H} (150.9 MHz, CDCl_3_) δ 164.3, 144.1, 135.0 (q, ^2^*J*_C-F_ = 32.4 Hz), 133.0, 130.0, 130.0 (q, ^2^*J*_C-F_ = 32.4 Hz), 125.9, 125.8 (q, ^3^*J*_C-F_ = 3.3 Hz), 125.6 (q, ^3^*J*_C-F_ = 3.3 Hz), 123.5, 123.9 (2 × q, ^1^*J*_C-F_ = 272.4 Hz), 74.7, 68.9, 56.4, 51.5, 40.6, 38.4, 27.3, 22.9, 22.0, 20.1 ppm. HRMS (ESI) *m*/*z* calcd for C_27_H_32_ F_6_NO_3_S [M + H]^+^: 564.2002, found 564.1997.

## Supporting Information Available

NMR and X-ray data for dimethylcyclopentanone reactions; energies and geometries of computed structures (PDF)

## Accession Codes

CCDC 2044260 and 2044371 contain the supplementary crystallographic data for this paper. These data can be obtained free of charge via www.ccdc.cam.ac.uk/data_request/cif, or by emailing data_request@ccdc.cam.ac.uk, or by contacting The Cambridge Crystallographic Data Centre, 12 Union Road, Cambridge CB2 1EZ, UK; fax: +44 1223 336033.
