# Supplementary material for: Mechanism and Origins of Stereoselectivity of the Aldol-Tishchenko Reaction of Sulfinimines
Source: J Org Chem. 2021 Feb 15;86(5):4296–303. doi: 10.1021/acs.joc.0c02862 (PMC8279497; doi:10.1021/acs.joc.0c02862)
Supplement: Supplementary file 1 — jo0c02862_si_001.pdf [file jo0c02862_si_001.pdf]

Supporting Information

Mechanism and Origins of Stereoselectivity of the Aldol-Tishchenko Reaction of Sulfinimines

Aneta Turlik,[a] Kaori Ando,[b] Pamela Mackey,[c] Emma Alcock,[c] Mark Light,[d] Gerard P. McGlacken,[c]* K. N. Houk[a]*

[a] Department of Chemistry and Biochemistry, University of California, Los Angeles, California 90095-1569, United States

[b] Department of Chemistry and Biomolecular Science, Faculty of Engineering, Gifu University, Yanagido 1-1, Gifu 501-1193, Japan

[c] School of Chemistry and Analytical and Biological Chemistry Research Facility, University College Cork, Cork, Ireland

[d] School of Chemistry, University of Southampton, Southampton, SO17 1BJ, UK

Table of Contents

1. Synthesis of dimethylcyclopentanone-derived sulfinimines 2
   1.1 NMR Spectra 3
   1.2 X-ray data........ 8
2. DFT calculations 10
   2.1 Summary of Energies........ 10
   2.2 Coordinates 11
3. References for supporting information 43

1. Synthesis of dimethylcyclopentanone-derived sulfinimines

Compound	Yield	*dr*
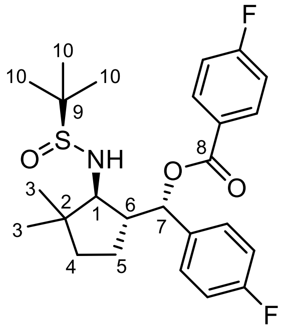	60%	91:9
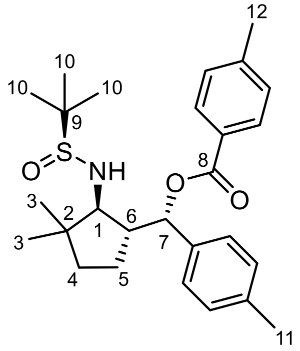	52%	88:12
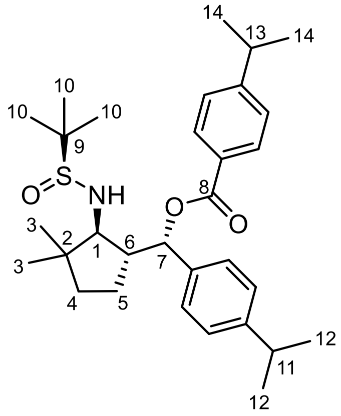	61%	91:9
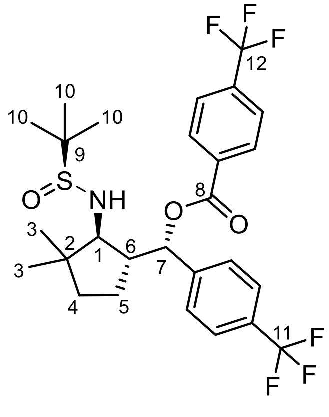	43%	>99:1

1.1 NMR Spectra

(*S*)-*N*-(2,2-dimethylcyclopentylidene)-2-methylpropane-2-sulfinamide, 9

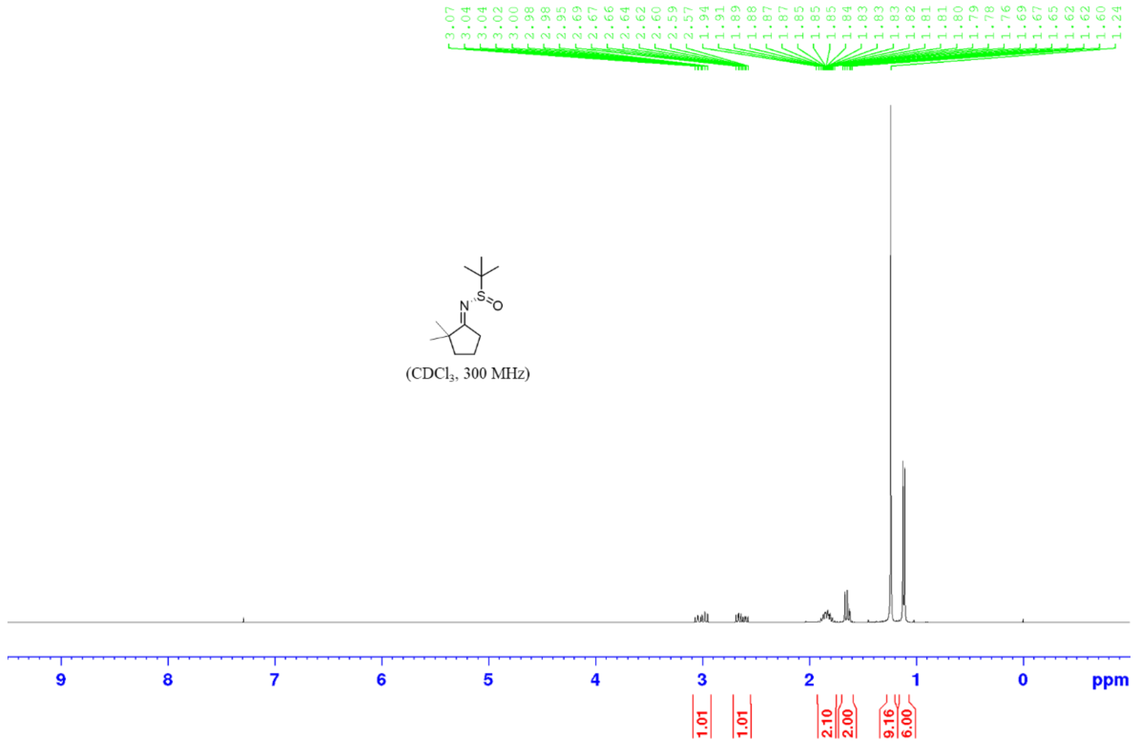

(*S*)-*N*-(2,2-dimethylcyclopentylidene)-2-methylpropane-2-sulfinamide, 9

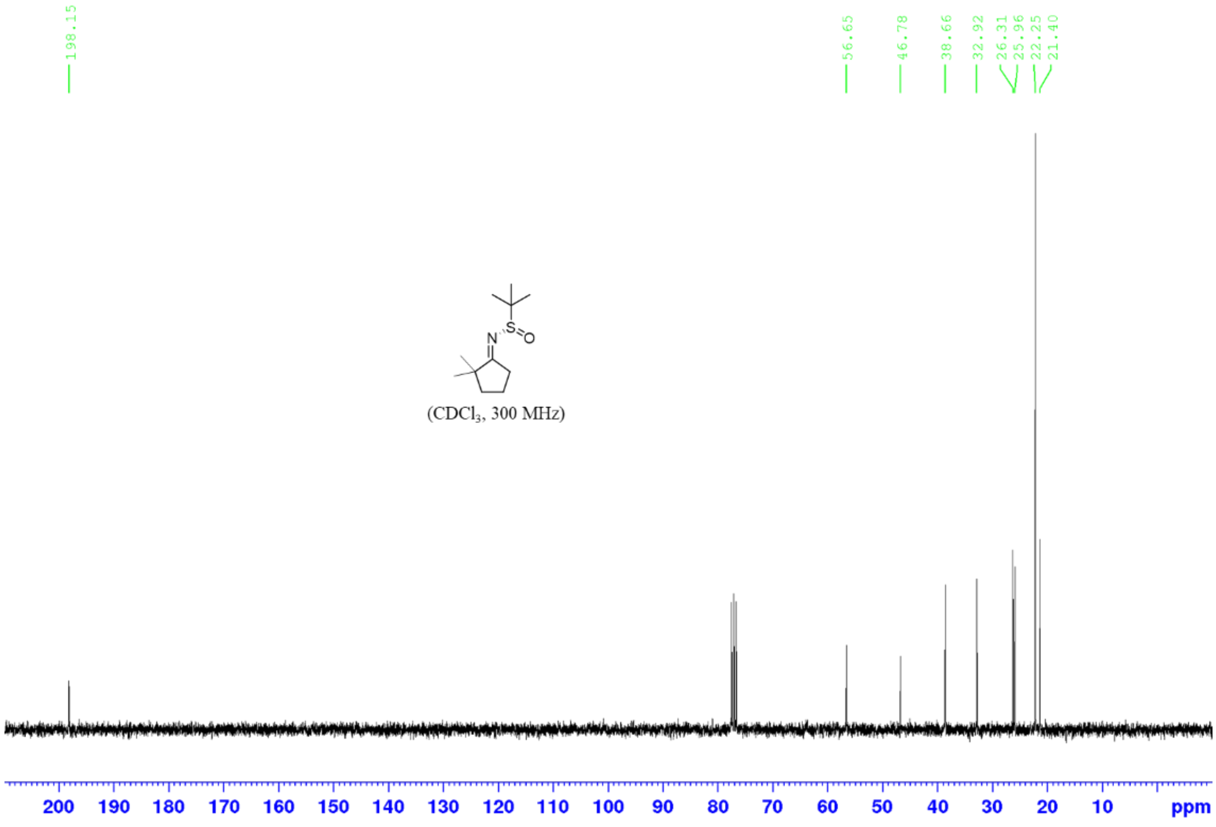

(*S*)-((1*R*,2*S*)-2-(((*S*)-*tert*-butylsulfinyl)amino)-3,3-dimethylcyclopentyl)(4-fluorophenyl)methyl 4-fluorobenzoate, 12

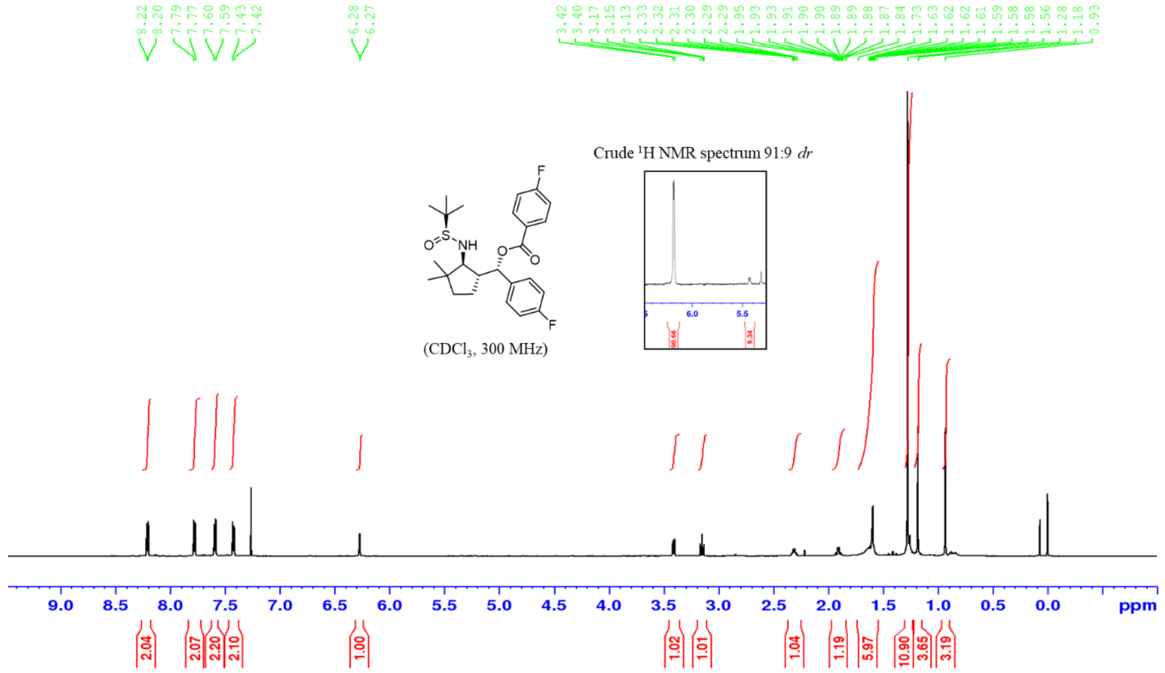

(*S*)-((1*R*,2*S*)-2-(((*S*)-*tert*-butylsulfinyl)amino)-3,3-dimethylcyclopentyl)(4-fluorophenyl)methyl 4-fluorobenzoate, 12

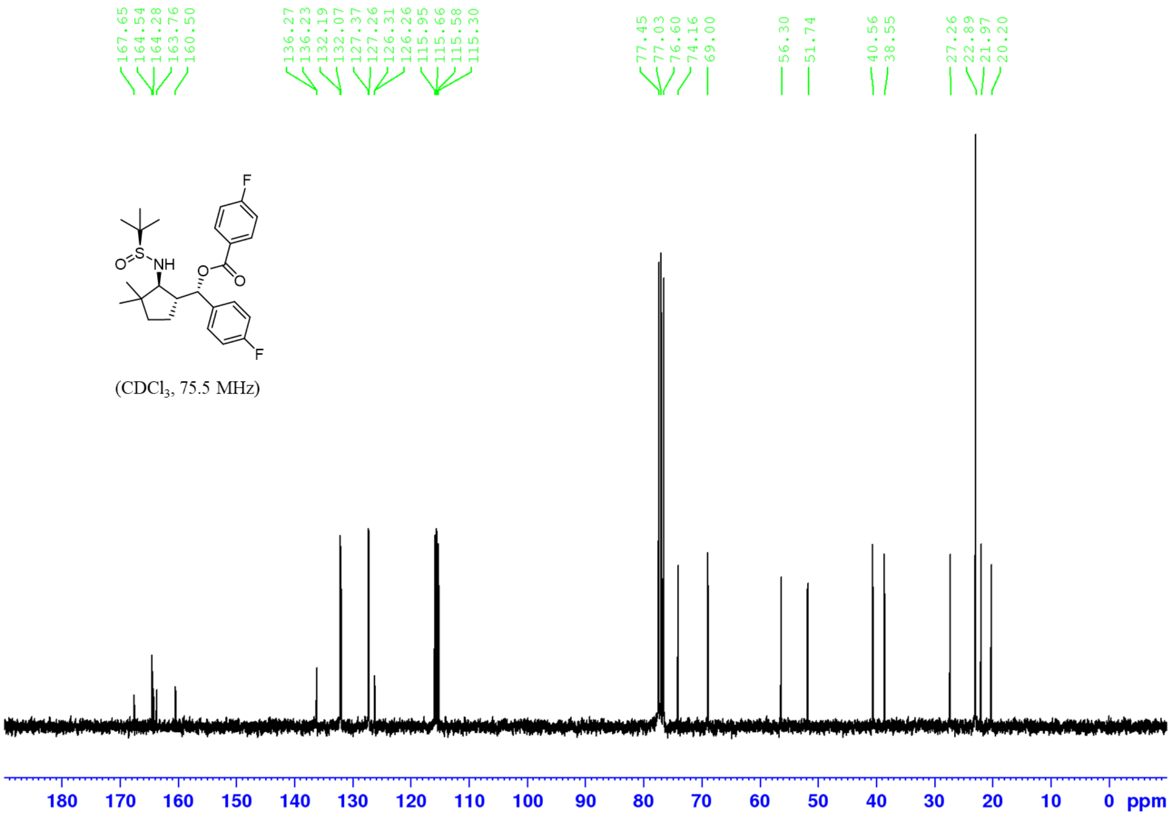

(*S*)-((1*R*,2*S*)-2-(((*S*)-*tert*-butylsulfinyl)amino)-3,3-dimethylcyclopentyl)(4-isopropylphenyl)methyl 4-isopropylbenzoate, 13

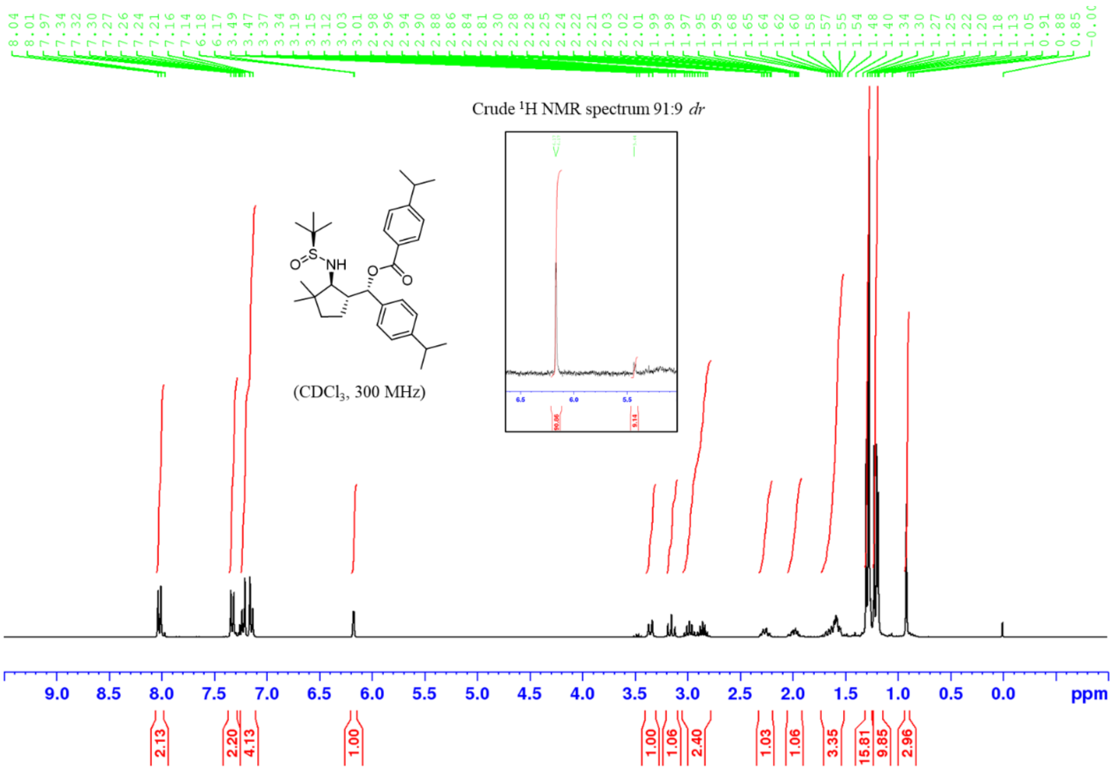

(*S*)-((1*R*,2*S*)-2-(((*S*)-*tert*-butylsulfinyl)amino)-3,3-dimethylcyclopentyl)(4-isopropylphenyl)methyl 4-isopropylbenzoate, 13

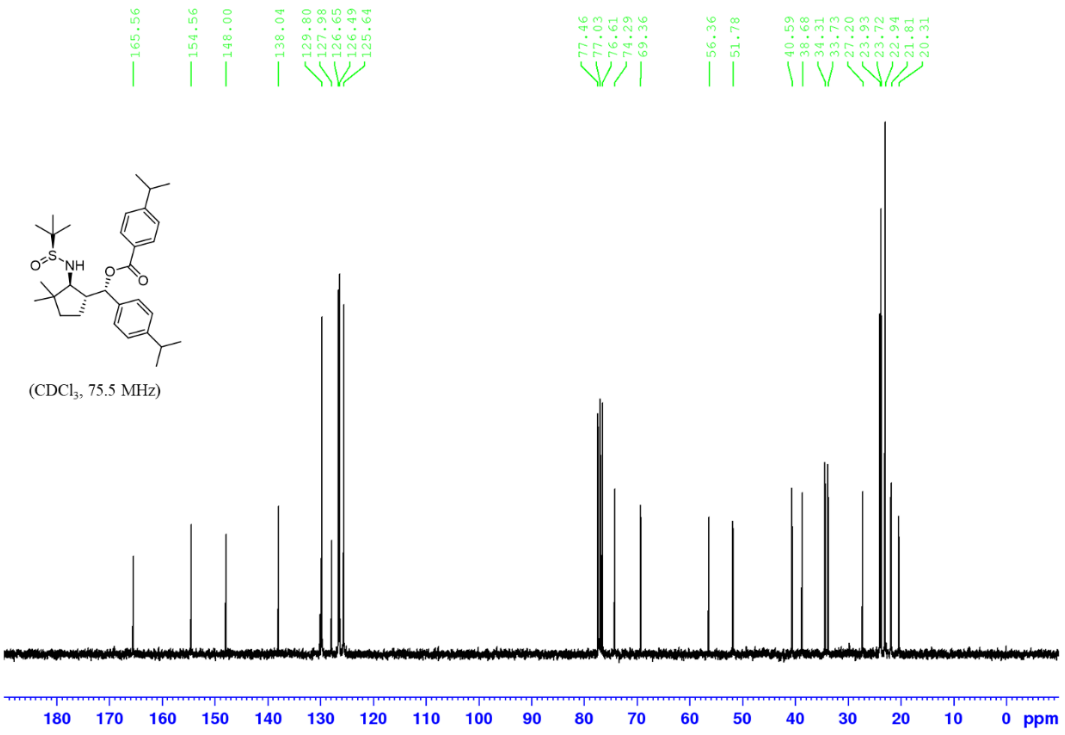

(*S*)-((1*R*,2*S*)-2-(((*S*)-*tert*-butylsulfinyl)amino)-3,3-dimethylcyclopentyl)(*p*-tolyl)methyl 4-methylbenzoate, 14

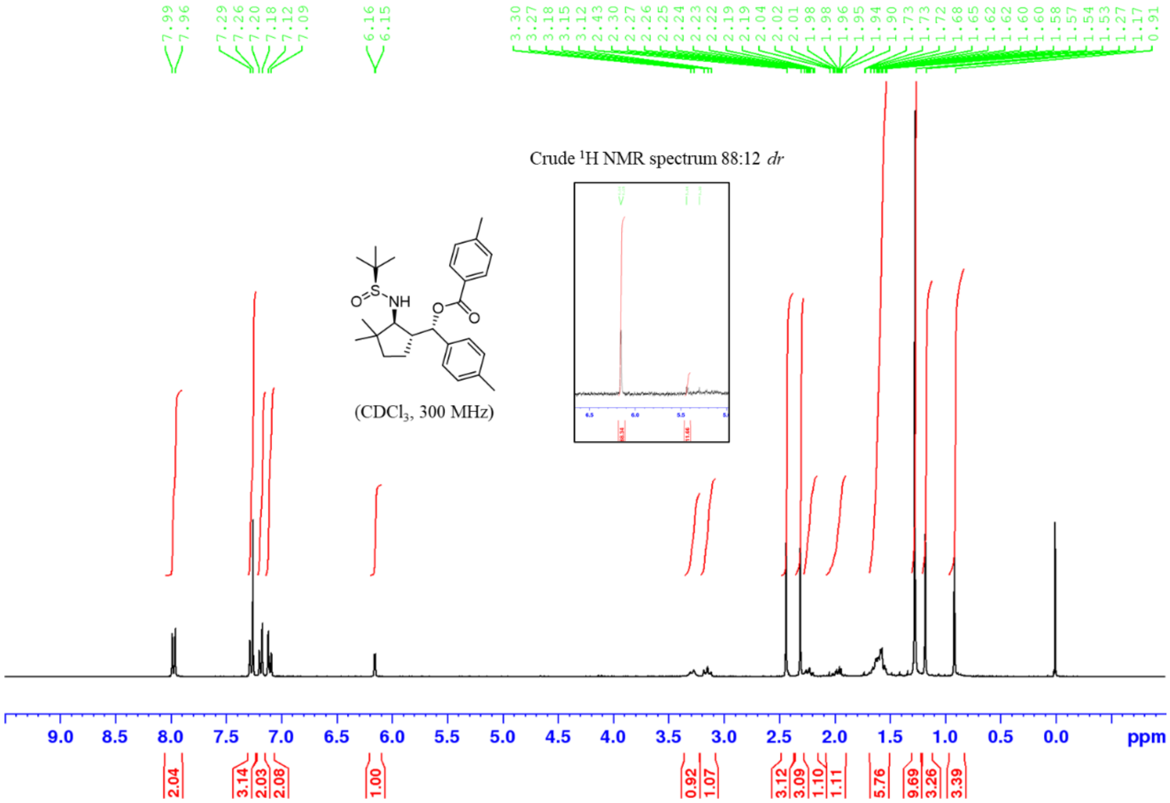

(*S*)-((1*R*,2*S*)-2-(((*S*)-*tert*-butylsulfinyl)amino)-3,3-dimethylcyclopentyl)(*p*-tolyl)methyl 4-methylbenzoate, 14

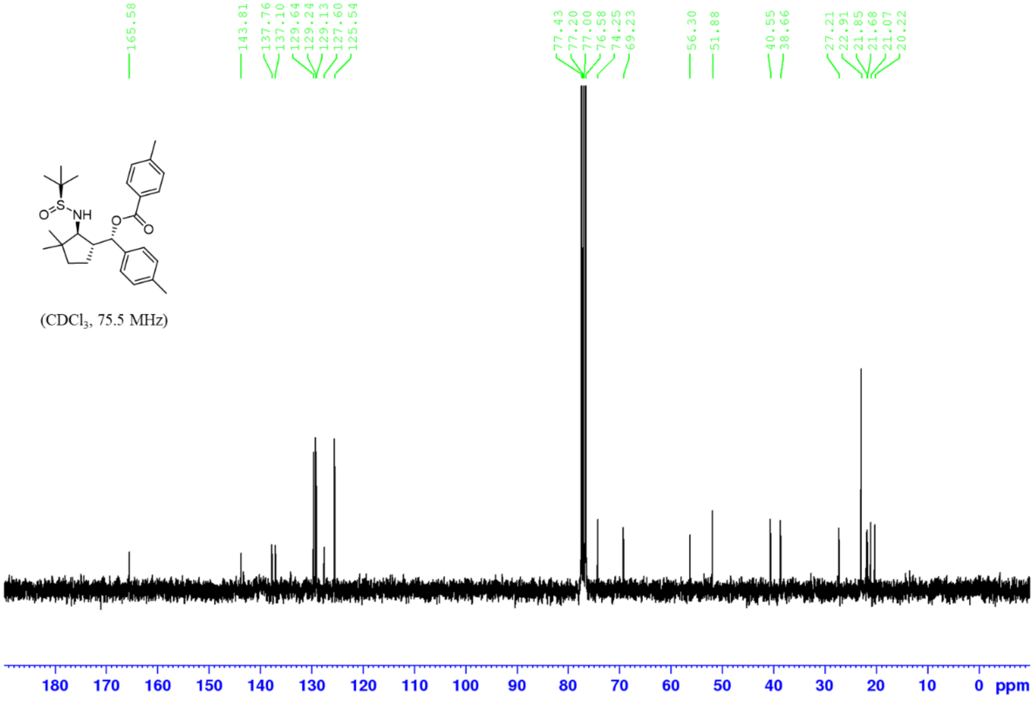

(*S*)-((1*R*,2*S*)-2-(((*S*)-*tert*-butylsulfinyl)amino)-3,3-dimethylcyclopentyl)(4-(trifluoromethyl)phenyl)methyl 4-(trifluoromethyl)benzoate, 15

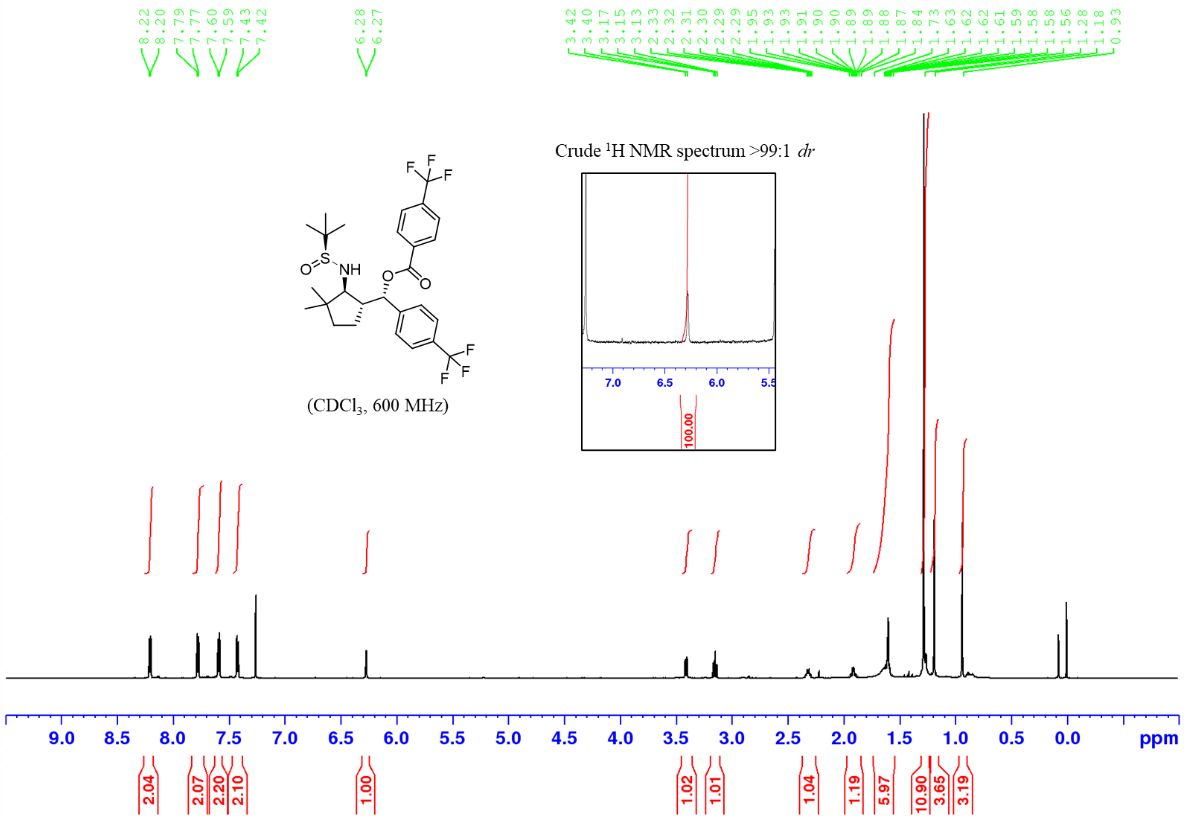

(*S*)-((1*R*,2*S*)-2-(((*S*)-*tert*-butylsulfinyl)amino)-3,3-dimethylcyclopentyl)(4-(trifluoromethyl)phenyl)methyl 4-(trifluoromethyl)benzoate, 15

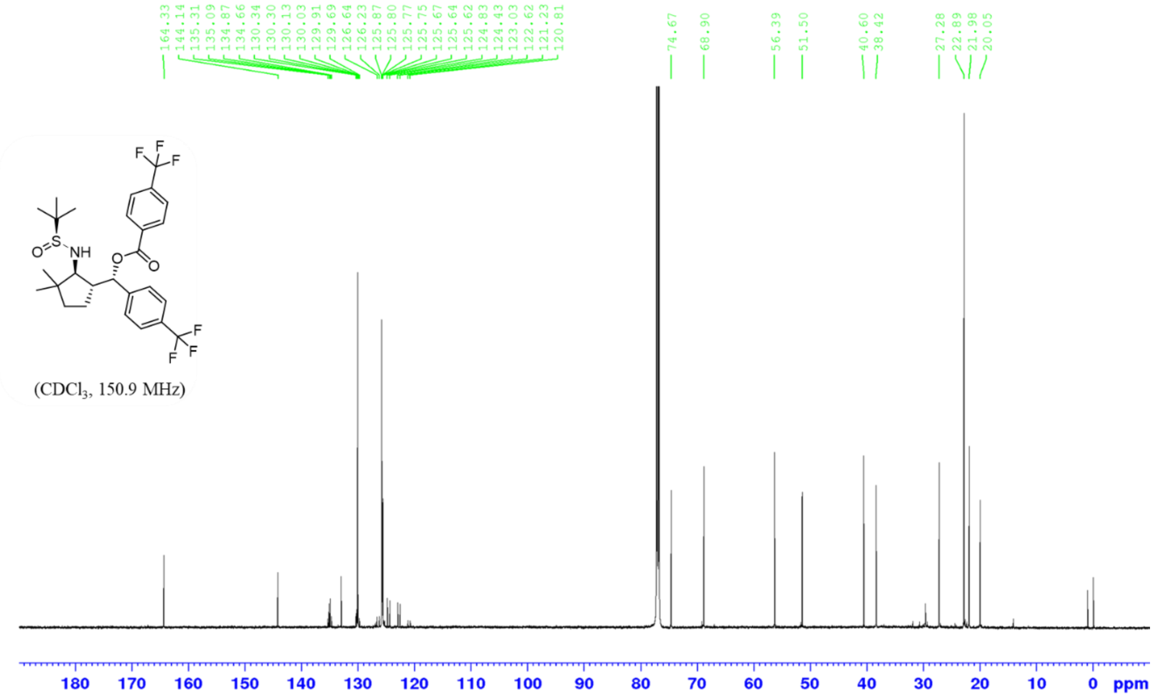

1.2 X-ray data

Compound 12

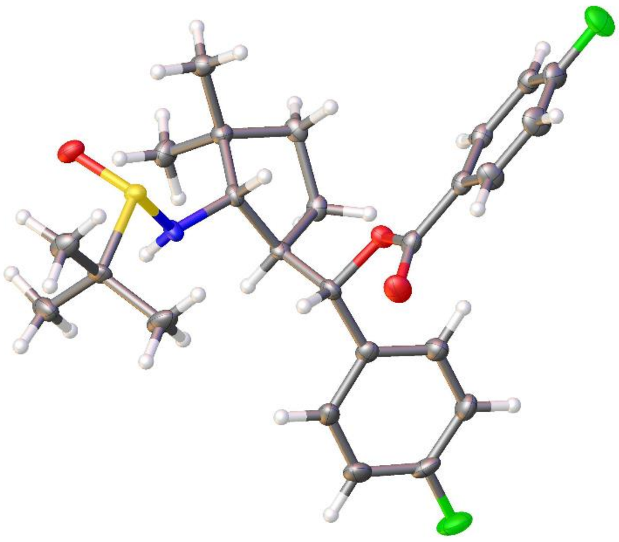

Figure 1: Thermal ellipsoids drawn at the 50% probability level. Second molecule omitted for clarity.

Experimental. Single clear colourless prism crystals of **12** recrystallised from $CHCl_3$ by slow evaporation. A suitable crystal with dimensions 0.24 × 0.15 × 0.04 mm^3 was selected and mounted on a MITIGEN holder with silicon oil on a Rigaku AFC12 FRE-HF diffractometer. The crystal was kept at a steady T = 100(2) K during data collection. The structure was solved with the ShelXT 2018/2 (Sheldrick, 2018) solution program using dual methods and by using Olex2 (Dolomanov et al., 2009) as the graphical interface. The model was refined with ShelXL 2018/3 (Sheldrick, 2015) using full matrix least squares minimisation on $\boldsymbol{F}^2$.

Crystal Data. $C_{25}H_{31}F_2NO_3S$, M_r = 463.57, orthorhombic, $P2_12_12_1$ (No. 19), a = 10.7171(2) Å, b = 18.7688(4) Å, c = 24.6349(6) Å, $\alpha = \beta = \gamma$ = 90°, V = 4955.24(19) Å^3, T = 100(2) K, Z = 8, Z' = 2, μ(Mo K_α) = 0.171, 50204 reflections measured, 12533 unique (R_{int} = 0.0593) which were used in all calculations. The final wR_2 was 0.1189 (all data) and R_1 was 0.0663 (I≥2 σ(I)).

Compound 14

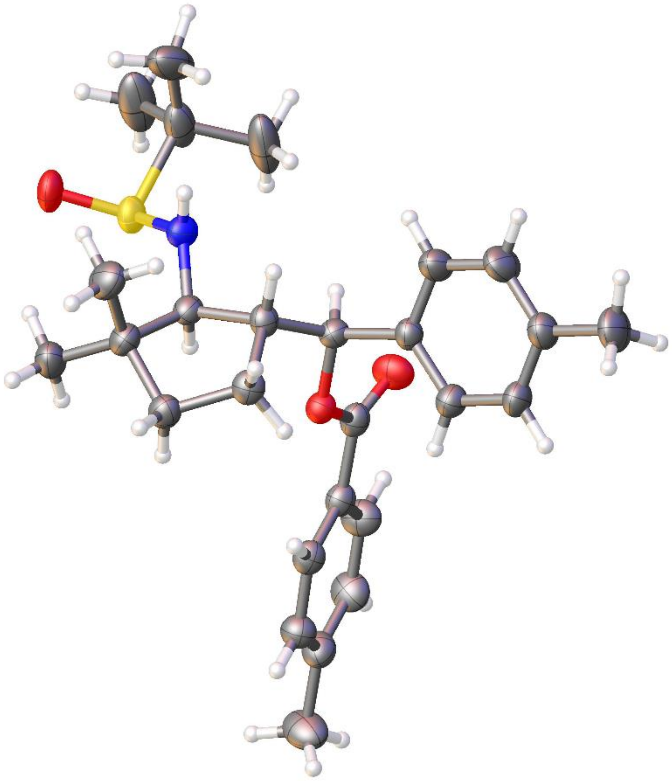

Figure 2: Thermal ellipsoids drawn at the 50% probability level.

Experimental. Single clear colourless prism crystals of **14** recrystallised from $CHCl_3$ by slow evaporation. A suitable crystal with dimensions 0.36 × 0.18 × 0.04 mm³ was selected and mounted on a MITIGEN holder with silicon oil on a Rigaku AFC12 FRE-HF diffractometer. The crystal was kept at a steady T = 100(2) K during data collection. The structure was solved with the ShelXT 2018/2 (Sheldrick, 2018) solution program using dual methods and by using Olex2 (Dolomanov et al., 2009) as the graphical interface. The model was refined with ShelXL 2018/3 (Sheldrick, 2015) using full matrix least squares minimisation on $\boldsymbol{F}^2$.

Crystal Data. $C_{27}H_{37}NO_3S$, M_r = 455.63, orthorhombic, $P2_12_12_1$ (No. 19), a = 11.12360(10) Å, b = 18.9136(2) Å, c = 25.0705(2) Å, $\alpha = \beta = \gamma = 90°$, V = 5274.52(8) Å³, T = 100(2) K, Z = 8, Z' = 2, μ(Mo K_α) = 0.149, 57089 reflections measured, 12429 unique (R_{int} = 0.0524) which were used in all calculations. The final wR_2 was 0.1398 (all data) and R_1 was 0.0538 (I≥2 σ(I)).

2. DFT calculations

Density functional theory (DFT) calculations were performed with Gaussian 16.[1] Spartan'16 and CREST were used for conformational searches.[2,3] Molecular geometry optimizations and frequency calculations were performed using the B3LYP functional,[4] augmented with Grimme's D3 empirical dispersion term,[5] and the 6-31G(d) basis set. Frequency calculations confirmed the optimized structures as minima (zero imaginary frequencies) or transition state structures (one imaginary frequency) on the potential energy surface. Intrinsic reaction coordinate (IRC) calculations were performed in order to connect the transition states to the reactants and the products. Single point energies were calculated using M06-2X-D3/6-311+G(d,p),[6] and a quasi-harmonic correction was applied using the GoodVibes program.[7] 3D renderings of optimized structures were generated using PyMol 2.3.2.[8] GaussView 6.0.16 was used to generate initial structures.[9]

2.1 Summary of Energies

Structure	E_SPC (au)	qh-H_SPC (au)	T.qh-S (au)	qh-G(T)_SPC (au)	qh-G(T)_SPC (kcal/mol)
**Figure 1b: Solvation models**					
**THF**	-232.41	-232.29	0.033873	-232.32	-145782.8
**SM-2-0**	-1732.91	-1732.38	0.090622	-1732.47	-1087141.0
**SM-2-1**	-1965.35	-1964.70	0.103891	-1964.80	-1232931.0
**SM-2-2**	-2197.78	-2197.01	0.117124	-2197.13	-1378717.5
**Figure 1c: Solvation model TSs**					
**Unsolvated:**					
**SM-2-0**	-1732.91	-1732.38	0.090622	-1732.47	-1087141.0
**TS-2a-0**	-1732.89	-1732.36	0.088728	-1732.45	-1087126.4
**Mono-solvated:**					
**SM-2-1**	-1965.35	-1964.70	0.103891	-1964.80	-1232931.0
**TS-2a-1**	-1965.33	-1964.68	0.103170	-1964.78	-1232917.1
**Di-solvated:**					
**SM-2-2**	-2197.78	-2197.01	0.117124	-2197.13	-1378717.5
**TS-2a-2**	-2197.76	-2196.99	0.114125	-2197.10	-1378703.0
**Figure 2: Propiophenone: Three lowest-energy diastereomers**					
**TS-2a**	-2197.76	-2196.99	0.114125	-2197.10	-1378703.0
**TS-2b**	-2197.76	-2196.99	0.114384	-2197.10	-1378702.4
**TS-2c**	-2197.76	-2196.99	0.114255	-2197.10	-1378702.1
**Figure 3: Propiophenone: *E* vs *Z***					
**TS-2a-*Z***	-2197.76	-2196.99	0.114125	-2197.10	-1378703.0

**TS-2a-*E***	-2197.75	-2196.98	0.113659	-2197.09	-1378696.6
**Figure 4: Acetophenone**					
**TS-3a-*E***	-2010.88	-2010.07	0.112871	-2010.18	-1261407.1
**TS-3b**	-2010.87	-2010.06	0.113863	-2010.18	-1261404.7
**TS-3a-*Z***	-2010.86	-2010.05	0.113151	-2010.16	-1261394.5
**Figure 5: Effect of substitution at C2**					
**TS-4a**	-2158.46	-2157.72	0.111638	-2157.83	-1354057.5
**TS-4b**	-2158.46	-2157.71	0.111979	-2157.83	-1354055.4
**Figure 6: Dimethylcyclopentanone**					
**TS-5a**	-2122.78	-2121.99	0.110372	-2122.10	-1331639.1
**TS-5b**	-2122.77	-2121.99	0.111320	-2122.10	-1331636.5
**TS-5c**	-2122.74	-2121.96	0.111973	-2122.07	-1331618.8

2.2 Coordinates

Fig1a-THF

C	-1.163286	-0.431165	0.136482
C	1.165610	-0.428015	-0.132024
C	-0.734158	0.994254	-0.232654
H	-1.522503	-0.476296	1.176867
H	-1.953348	-0.827259	-0.512470
C	0.730232	0.997645	0.230631
H	1.534908	-0.475083	-1.168579
H	1.950619	-0.820232	0.525556
H	-0.789258	1.141236	-1.318152
H	-1.348801	1.762193	0.247221
H	1.342385	1.765602	-0.252357
H	0.784368	1.150168	1.315412
O	0.001405	-1.252080	-0.003513

Fig1a-SM-0THF

C	1.484592	0.806822	-0.826122
C	0.129577	1.303003	-1.329809
C	-0.997081	0.712033	-0.418659
C	-0.944096	0.463172	2.042721
H	0.093106	2.391672	-1.217513
H	-0.786335	-0.357618	-0.305061
O	-0.933767	1.365606	0.839793
O	0.189751	-0.217589	2.163917
Li	1.861611	-0.429547	2.063148
H	-1.096157	1.228735	2.827447
C	2.377400	1.759160	-0.104554

C	3.733500	1.850986	-0.460282
C	1.869124	2.604356	0.896065
C	4.573714	2.751520	0.189590
H	4.128037	1.231944	-1.260897
C	2.723243	3.483094	1.562707
H	0.817014	2.531384	1.151712
C	4.073362	3.560486	1.212982
H	5.617868	2.820865	-0.102000
H	2.327499	4.116855	2.351587
H	4.731528	4.255407	1.727501
S	3.117818	-1.266642	-0.509792
O	3.255219	-1.070965	1.012581
C	2.395300	-2.993139	-0.717594
C	1.082763	-3.118218	0.054438
H	0.325213	-2.428703	-0.322733
H	0.706474	-4.142771	-0.052810
H	1.225027	-2.922435	1.121019
C	3.471009	-3.926769	-0.146169
H	4.423016	-3.827717	-0.681149
H	3.644279	-3.725756	0.915021
H	3.133551	-4.964006	-0.250737
C	2.201509	-3.209733	-2.221975
H	3.138318	-3.076707	-2.776274
H	1.453142	-2.524034	-2.626826
H	1.858793	-4.237780	-2.388238
C	-2.374904	0.882112	-1.030916
C	-3.003603	2.132782	-1.007449
C	-3.039427	-0.200829	-1.612088
C	-4.272055	2.297600	-1.561058
H	-2.500146	2.963053	-0.520593
C	-4.312523	-0.041592	-2.161892
H	-2.571017	-1.181922	-1.601708
C	-4.930954	1.209291	-2.141135
H	-4.753098	3.272259	-1.532656
H	-4.825385	-0.896864	-2.594392
H	-5.923940	1.335063	-2.565053
C	-2.203320	-0.405405	1.952680
C	-3.471255	0.186716	1.962199
C	-2.102528	-1.788989	1.799080
C	-4.618852	-0.587883	1.806347
H	-3.549938	1.266754	2.058304
C	-3.249343	-2.570633	1.636493
H	-1.112530	-2.232806	1.810872
C	-4.510311	-1.971639	1.636750
H	-5.597141	-0.113762	1.801935
H	-3.158998	-3.647686	1.512490
H	-5.404159	-2.577349	1.508587
C	-0.053634	0.945137	-2.814223

H	-0.969655	1.398554	-3.199670
H	0.793122	1.308354	-3.406851
H	-0.121298	-0.138282	-2.947782
N	1.743860	-0.426692	-1.112198

Fig1a-SM-1THF

C	0.680993	0.840428	1.578110
C	-0.699885	0.436000	2.095505
C	-1.560341	-0.032154	0.877741
C	-0.819787	-1.257946	-1.141308
H	-0.587641	-0.424844	2.763528
H	-1.507104	0.763353	0.125275
O	-0.982990	-1.222834	0.365798
O	0.063459	-0.371897	-1.571023
Li	1.777382	-0.052041	-1.405112
H	-0.502062	-2.318559	-1.237787
C	1.833956	-0.075095	1.834567
C	3.046629	0.454153	2.306203
C	1.699360	-1.468329	1.714620
C	4.106649	-0.386409	2.639954
H	3.154525	1.526579	2.439138
C	2.768386	-2.304027	2.035021
H	0.760092	-1.865081	1.343065
C	3.971730	-1.769883	2.501686
H	5.033543	0.038583	3.014865
H	2.656230	-3.379822	1.927660
H	4.797414	-2.426664	2.762821
S	2.069756	2.579338	0.122818
O	2.684329	1.482914	-0.758388
C	1.072857	3.690211	-1.033309
C	-0.142606	2.959031	-1.606177
H	-0.919156	2.836798	-0.848649
H	-0.552686	3.557471	-2.429513
H	0.100822	1.962199	-1.982962
C	2.065588	4.082189	-2.134558
H	2.965826	4.557840	-1.726584
H	2.369304	3.208639	-2.717808
H	1.582670	4.799877	-2.807239
C	0.658650	4.898937	-0.186608
H	1.526395	5.439501	0.209250
H	0.021580	4.593541	0.649064
H	0.087452	5.592096	-0.815434
C	-3.008736	-0.266295	1.265647
C	-3.358523	-1.395941	2.015343
C	-4.012117	0.622426	0.871827
C	-4.686391	-1.630042	2.367239
H	-2.581731	-2.104825	2.288129
C	-5.344667	0.388460	1.215947

H	-3.751657	1.483123	0.261102
C	-5.685193	-0.736860	1.967550
H	-4.946578	-2.513752	2.944957
H	-6.117046	1.078438	0.885823
H	-6.722675	-0.923355	2.233125
C	-2.222863	-1.114411	-1.740983
C	-3.213738	-2.060968	-1.456082
C	-2.548506	-0.010035	-2.529451
C	-4.511562	-1.899767	-1.935965
H	-2.967022	-2.908214	-0.820867
C	-3.849559	0.159677	-3.010549
H	-1.765893	0.706867	-2.753972
C	-4.835221	-0.782557	-2.712412
H	-5.275647	-2.634253	-1.693391
H	-4.093857	1.027627	-3.619365
H	-5.849122	-0.650527	-3.082552
C	-1.355636	1.588405	2.872272
H	-2.299753	1.256759	3.311009
H	-0.698752	1.936264	3.677045
H	-1.558284	2.435840	2.211228
N	0.715398	1.990625	0.995066
O	3.050167	-1.443228	-1.679933
C	4.416412	-1.422672	-1.195373
C	2.561016	-2.799298	-1.737800
C	4.812443	-2.892232	-0.984649
H	5.028272	-0.932255	-1.960385
H	4.452796	-0.829430	-0.277495
C	3.819556	-3.650517	-1.883112
H	2.020950	-3.030015	-0.809826
H	1.862790	-2.863007	-2.575326
H	4.664076	-3.174269	0.062526
H	5.858664	-3.080680	-1.242844
H	3.664943	-4.688825	-1.574477
H	4.159201	-3.647207	-2.925365

Fig1a-SM-2THF

C	0.040047	-1.650196	1.475339
C	-1.436681	-2.020003	1.299941
C	-2.096125	-0.983668	0.336457
C	-0.988668	0.110645	-1.572278
H	-1.500387	-2.999931	0.815388
H	-1.848184	0.010427	0.726396
O	-1.533183	-1.155902	-0.952992
O	0.012542	0.627766	-0.887697
Li	1.736096	0.548111	-0.477698
H	-0.715571	-0.306308	-2.566993
C	1.067412	-2.478041	0.780318
C	2.256627	-2.830133	1.438888

C	0.822780	-2.998325	-0.501680
C	3.184689	-3.671692	0.829850
H	2.446682	-2.475734	2.447306
C	1.757619	-3.831259	-1.111261
H	-0.083931	-2.700649	-1.016687
C	2.938939	-4.173219	-0.450278
H	4.093199	-3.944474	1.359950
H	1.559712	-4.218307	-2.107472
H	3.659860	-4.834705	-0.924127
S	1.735980	0.090817	2.596171
O	2.519476	0.403967	1.316056
C	1.010768	1.740799	3.147371
C	0.171393	2.342670	2.022519
H	-0.703931	1.729546	1.803995
H	-0.162930	3.343918	2.323615
H	0.749844	2.431745	1.101383
C	2.236954	2.609539	3.458063
H	2.859948	2.168948	4.245782
H	2.854502	2.752514	2.567381
H	1.900522	3.592600	3.807028
C	0.183931	1.475475	4.408737
H	0.783585	1.003089	5.196243
H	-0.672800	0.834109	4.189443
H	-0.185426	2.432040	4.797878
C	-3.604776	-1.132446	0.272100
C	-4.175770	-2.196997	-0.436013
C	-4.445416	-0.207166	0.896332
C	-5.560371	-2.337082	-0.511362
H	-3.521187	-2.893571	-0.952165
C	-5.833087	-0.339903	0.818794
H	-4.008791	0.639791	1.419934
C	-6.394611	-1.407771	0.117619
H	-5.991843	-3.165693	-1.067859
H	-6.474202	0.396923	1.296183
H	-7.474770	-1.511570	0.052524
C	-2.185695	1.056762	-1.752605
C	-3.289519	0.677520	-2.524659
C	-2.207822	2.291470	-1.101831
C	-4.397408	1.514746	-2.640578
H	-3.286214	-0.297287	-3.006018
C	-3.317421	3.134013	-1.209712
H	-1.342082	2.563595	-0.506848
C	-4.415845	2.747289	-1.979355
H	-5.254057	1.203118	-3.233397
H	-3.325475	4.091431	-0.692616
H	-5.282266	3.398920	-2.063999
C	-2.149113	-2.095297	2.659470
H	-3.169649	-2.463751	2.528703

H	-1.617746	-2.769989	3.339530
H	-2.195184	-1.109072	3.130181
N	0.233679	-0.657446	2.277085
O	2.874616	-0.705450	-1.457927
C	4.291620	-0.675296	-1.267120
C	2.657293	-0.811988	-2.867287
C	4.850051	-1.694695	-2.277435
H	4.665485	0.339813	-1.473788
H	4.477094	-0.920069	-0.220384
C	3.740280	-1.789032	-3.362411
H	1.631880	-1.158480	-3.010942
H	2.768253	0.180822	-3.329920
H	4.995750	-2.664878	-1.795197
H	5.812772	-1.372284	-2.685651
H	3.341168	-2.805836	-3.411960
H	4.100256	-1.520438	-4.360141
O	2.507852	2.268046	-1.191617
C	3.412923	3.135641	-0.502462
C	1.637343	3.103590	-1.973099
C	2.600637	4.402127	-0.151019
H	3.786154	2.583343	0.363020
H	4.258473	3.380269	-1.163414
C	1.360293	4.324922	-1.083554
H	2.152208	3.391348	-2.902669
H	0.752911	2.505196	-2.193766
H	3.191447	5.308147	-0.317121
H	2.297565	4.395362	0.899665
H	1.217993	5.235196	-1.673455
H	0.449576	4.160114	-0.501542

Fig1b-TS-2a-0THF

C	-0.479656	-0.398031	0.373801
C	0.858157	-1.087406	0.684935
C	1.885984	-0.937830	-0.490649
C	0.930468	1.092141	-1.243715
H	1.299966	-0.569675	1.543648
H	1.467022	-1.435024	-1.375385
O	2.106560	0.438509	-0.769968
O	0.414475	0.656171	-2.349153
Li	-0.942128	-0.435425	-2.665783
H	0.170719	0.779741	-0.333133
C	-1.097731	0.445258	1.466278
C	-1.187331	-0.046784	2.775921
C	-1.606253	1.722578	1.186631
C	-1.787373	0.715356	3.779608
H	-0.791813	-1.028324	3.015509
C	-2.198073	2.485637	2.191163
H	-1.514315	2.128248	0.183534

C	-2.294821	1.982839	3.490404
H	-1.851850	0.318160	4.789083
H	-2.575140	3.477302	1.957937
H	-2.755458	2.578302	4.273867
S	-2.816852	-0.537283	-0.876884
O	-2.814972	-0.438557	-2.422557
C	-3.688490	-2.165639	-0.509357
C	-3.072124	-3.278757	-1.354501
H	-2.024233	-3.440942	-1.088662
H	-3.625371	-4.210238	-1.184276
H	-3.132190	-3.031452	-2.418945
C	-5.153800	-1.918523	-0.892076
H	-5.594869	-1.106562	-0.301900
H	-5.244352	-1.667121	-1.953201
H	-5.736125	-2.827846	-0.701410
C	-3.531116	-2.418170	0.992797
H	-3.890327	-1.571674	1.589853
H	-2.485689	-2.604342	1.253410
H	-4.116989	-3.301422	1.273559
C	3.221303	-1.555285	-0.142113
C	4.092208	-0.900856	0.738245
C	3.591871	-2.798730	-0.663069
C	5.307298	-1.483974	1.094485
H	3.817423	0.076112	1.124002
C	4.806088	-3.387719	-0.304125
H	2.925808	-3.308415	-1.355579
C	5.666517	-2.731596	0.577070
H	5.977542	-0.963299	1.773562
H	5.081404	-4.353981	-0.718619
H	6.614291	-3.185390	0.854430
C	1.069719	2.587843	-1.047604
C	1.758774	3.112689	0.052059
C	0.411867	3.452707	-1.927239
C	1.801104	4.490480	0.260349
H	2.258504	2.434468	0.735921
C	0.452353	4.831772	-1.716509
H	-0.118735	3.026707	-2.772509
C	1.146408	5.353894	-0.622683
H	2.342744	4.892054	1.113084
H	-0.056137	5.499188	-2.407750
H	1.177272	6.427939	-0.457895
C	0.645421	-2.572706	1.026762
H	1.578032	-3.025666	1.372410
H	-0.108012	-2.698710	1.810573
H	0.295910	-3.117779	0.144938
N	-1.204308	-1.011952	-0.577192

Fig1b-TS-2a-1THF

C	-0.233668	-0.955403	-0.780544
C	1.128270	-0.750531	-1.464744
C	1.945888	0.402034	-0.786043
C	0.885534	0.102206	1.308059
H	1.710351	-1.667737	-1.320231
H	1.378359	1.334729	-0.906625
O	2.127155	0.124602	0.596973
O	0.186819	1.185935	1.310015
Li	-1.072913	1.859014	0.181682
H	0.294332	-0.718563	0.602198
C	-0.665896	-2.379229	-0.503599
C	-0.553773	-3.359240	-1.499734
C	-1.201726	-2.740031	0.741696
C	-0.983798	-4.665648	-1.261754
H	-0.133560	-3.104116	-2.466988
C	-1.624810	-4.046173	0.980715
H	-1.265370	-1.998873	1.532478
C	-1.521838	-5.012873	-0.021764
H	-0.892495	-5.412171	-2.046281
H	-2.026979	-4.308404	1.955243
H	-1.850991	-6.031459	0.164974
S	-2.718308	-0.080300	-0.464973
O	-2.937322	1.304643	0.179911
C	-3.594642	0.037497	-2.129479
C	-3.148564	1.310505	-2.847208
H	-2.082294	1.275381	-3.085489
H	-3.714318	1.417902	-3.780743
H	-3.337743	2.189785	-2.223704
C	-5.088362	0.087066	-1.782634
H	-5.409651	-0.814788	-1.248025
H	-5.315997	0.959217	-1.162074
H	-5.674662	0.157522	-2.706716
C	-3.238412	-1.228589	-2.912882
H	-3.477138	-2.138618	-2.350039
H	-2.174254	-1.249948	-3.163507
H	-3.811513	-1.249448	-3.847642
C	3.314599	0.559070	-1.407560
C	4.328158	-0.365726	-1.126233
C	3.579956	1.607187	-2.294197
C	5.580753	-0.246335	-1.726319
H	4.130664	-1.166683	-0.420289
C	4.832246	1.726703	-2.900638
H	2.800329	2.334163	-2.510770
C	5.835717	0.798871	-2.618575
H	6.361064	-0.967107	-1.495727
H	5.024208	2.547237	-3.587107
H	6.812596	0.891811	-3.085788
C	1.056054	-0.654296	2.608559

C	1.976129	-1.701394	2.733000
C	0.201548	-0.355566	3.674845
C	2.051204	-2.430755	3.919205
H	2.628938	-1.933554	1.898177
C	0.274086	-1.089066	4.859647
H	-0.510454	0.454785	3.554659
C	1.199480	-2.127987	4.985153
H	2.772917	-3.238472	4.012114
H	-0.390403	-0.849042	5.686019
H	1.256534	-2.699911	5.907853
C	0.957852	-0.471800	-2.968276
H	1.928323	-0.458503	-3.471180
H	0.332628	-1.231798	-3.447276
H	0.470215	0.495742	-3.119842
N	-1.102233	0.045863	-0.999512
C	-2.093887	4.409479	0.776508
C	0.190383	4.264974	1.003687
C	-1.868349	4.374603	2.300872
H	-2.954025	3.830398	0.435055
H	-2.171766	5.440393	0.402358
C	-0.325593	4.237683	2.450713
H	0.454304	5.281089	0.676439
H	1.025037	3.585694	0.826707
H	-2.261079	5.275688	2.781725
H	-2.376178	3.511570	2.740785
H	0.113683	5.038018	3.053678
H	-0.066706	3.277159	2.901704
O	-0.918757	3.797844	0.205868

Fig1b-TS-2a-2THF

C	0.847952	1.396268	0.550371
C	2.299124	0.860128	0.542479
C	2.360270	-0.612156	0.048257
C	0.396021	-0.411082	-1.232362
H	2.858988	1.448974	-0.192934
H	1.771534	-1.230359	0.730559
O	1.786765	-0.675811	-1.263247
O	-0.338197	-1.205568	-0.545414
Li	-1.829530	-1.227944	0.525192
H	0.431206	0.692115	-0.610274
C	0.759149	2.823508	0.038508
C	0.675644	3.879208	0.958623
C	0.875151	3.127553	-1.324624
C	0.684462	5.204761	0.524606
H	0.612458	3.655740	2.017832
C	0.875233	4.452701	-1.758970
H	0.958511	2.325853	-2.050283
C	0.777178	5.496893	-0.837085

H	0.618647	6.009004	1.252927
H	0.950841	4.667023	-2.821739
H	0.777193	6.529201	-1.176794
S	-1.486671	1.532519	1.649679
O	-2.454552	0.470611	1.071602
C	-1.725880	1.387261	3.510971
C	-1.417284	-0.034682	3.978077
H	-0.360624	-0.269764	3.836097
H	-1.656249	-0.121880	5.045803
H	-2.013263	-0.770446	3.430879
C	-3.200060	1.737282	3.750539
H	-3.440765	2.744982	3.390659
H	-3.853965	1.022800	3.242439
H	-3.413222	1.702319	4.825694
C	-0.794393	2.410516	4.167759
H	-0.998687	3.428375	3.814230
H	0.253644	2.169584	3.965473
H	-0.946974	2.398025	5.253928
C	3.771702	-1.137611	-0.037510
C	4.617259	-0.732835	-1.078116
C	4.264957	-2.009833	0.937938
C	5.932770	-1.190352	-1.137948
H	4.228602	-0.069258	-1.844775
C	5.583926	-2.465379	0.883525
H	3.610581	-2.334301	1.743816
C	6.421214	-2.056095	-0.155193
H	6.578717	-0.873046	-1.952647
H	5.953768	-3.143583	1.648189
H	7.446971	-2.412255	-0.202382
C	-0.098954	0.032170	-2.592291
C	0.680932	-0.050629	-3.749053
C	-1.390286	0.570645	-2.663051
C	0.159711	0.379728	-4.972713
H	1.685714	-0.453688	-3.683244
C	-1.902293	1.012266	-3.880953
H	-1.970716	0.653722	-1.748335
C	-1.129312	0.911288	-5.042673
H	0.764681	0.301767	-5.872612
H	-2.900784	1.440414	-3.924095
H	-1.527790	1.252718	-5.994504
C	2.938922	1.043310	1.924434
H	3.995855	0.762929	1.905249
H	2.863289	2.090602	2.237198
H	2.420771	0.431489	2.668545
N	0.086720	0.916655	1.563641
C	-3.146858	-2.377115	-1.847885
C	-4.645500	-1.306204	-0.379936
C	-4.195422	-1.633649	-2.702215

H	-3.245575	-3.466999	-1.926362
H	-2.118691	-2.092728	-2.080980
C	-4.794895	-0.602691	-1.725888
H	-4.585493	-0.629689	0.474484
H	-5.447670	-2.042507	-0.217792
H	-3.740341	-1.163695	-3.577817
H	-4.970980	-2.324870	-3.050349
H	-4.203471	0.317894	-1.719080
H	-5.832798	-0.345201	-1.958518
O	-3.380037	-1.984643	-0.471797
O	-1.490578	-2.728694	1.834712
C	-1.811541	-4.044693	1.355612
C	-0.094391	-2.758746	2.207775
C	-0.598704	-4.433682	0.509346
H	-1.943829	-4.724710	2.212022
H	-2.749173	-3.965705	0.803679
C	0.579927	-3.815939	1.299178
H	0.282646	-1.744405	2.072464
H	-0.022243	-3.033491	3.269193
H	-0.673727	-3.956964	-0.472197
H	-0.505739	-5.515481	0.371709
H	1.307512	-3.363794	0.622411
H	1.097342	-4.569606	1.902275

Fig2-TS-2b

C	-0.409848	0.718305	1.394185
C	-1.838606	0.116932	1.379487
C	-2.182031	-0.612345	0.042861
C	-0.691956	0.740880	-1.183920
H	-2.528026	0.965312	1.413690
H	-1.486082	-1.442680	-0.114159
O	-2.051072	0.324542	-1.024046
O	0.157539	-0.185851	-1.443276
Li	1.792667	-0.917884	-1.327383
H	-0.457961	1.118359	-0.016868
C	-0.326599	2.131960	1.934569
C	0.630967	2.460847	2.904271
C	-1.191548	3.140014	1.479041
C	0.709652	3.755907	3.417238
H	1.309525	1.689124	3.248022
C	-1.105524	4.435655	1.982375
H	-1.911427	2.919562	0.697725
C	-0.157282	4.748880	2.959353
H	1.452605	3.988465	4.176029
H	-1.774420	5.203534	1.603160
H	-0.092570	5.758911	3.355273
O	1.963462	-1.885243	0.297528

C	-3.597164	-1.138559	0.036793
C	-3.844735	-2.502687	0.218813
C	-4.681341	-0.264906	-0.112994
C	-5.153051	-2.988603	0.262565
H	-3.006364	-3.187665	0.324089
C	-5.988679	-0.747742	-0.074721
H	-4.489800	0.791436	-0.275688
C	-6.228627	-2.111282	0.116949
H	-5.330744	-4.051625	0.403411
H	-6.822255	-0.060976	-0.197581
H	-7.247941	-2.487231	0.146340
C	-0.642342	2.028211	-1.980626
C	-1.748483	2.879786	-2.086073
C	0.568275	2.395335	-2.580934
C	-1.645944	4.083279	-2.784535
H	-2.689944	2.582850	-1.636244
C	0.669919	3.602095	-3.273385
H	1.419885	1.727358	-2.501996
C	-0.435215	4.450622	-3.376195
H	-2.513129	4.733600	-2.869511
H	1.613572	3.879353	-3.736978
H	-0.354763	5.390399	-3.916619
C	-2.122172	-0.743533	2.620606
H	-1.756205	-0.247212	3.524658
H	-3.198612	-0.904558	2.725179
H	-1.645786	-1.724772	2.550999
N	0.742443	0.043783	1.544928
S	0.866699	-1.621035	1.353264
C	1.806949	-2.007534	2.950969
C	0.919105	-1.653030	4.145064
H	0.668188	-0.588563	4.149262
H	1.456222	-1.882715	5.073421
H	-0.011459	-2.230148	4.148002
C	2.087977	-3.514079	2.892458
H	1.158667	-4.095533	2.854048
H	2.690968	-3.763741	2.014806
H	2.638087	-3.819469	3.790903
C	3.107776	-1.204411	2.963125
H	3.679250	-1.453957	3.865704
H	2.904060	-0.130724	2.960155
H	3.715538	-1.444978	2.087964
O	1.834132	-2.262748	-2.801627
C	1.502473	-3.583306	-2.334172
C	0.844611	-1.935797	-3.804124
C	-0.025675	-3.575967	-2.220399

H	1.852265	-4.324410	-3.069408
H	2.018144	-3.728484	-1.383819
C	-0.470666	-2.605172	-3.346075
H	0.781386	-0.847899	-3.847479
H	1.187152	-2.327644	-4.772178
H	-0.310864	-3.177002	-1.242846
H	-0.454018	-4.578000	-2.322868
H	-1.163629	-1.857174	-2.957478
H	-0.950544	-3.133697	-4.175915
O	3.263877	0.358645	-1.571906
C	3.405613	1.223610	-0.408435
C	4.533227	-0.258378	-1.848274
C	4.763715	0.867415	0.233151
H	3.357518	2.265956	-0.742874
H	2.559320	1.016263	0.251199
C	5.163023	-0.443914	-0.470540
H	4.327999	-1.185389	-2.390451
H	5.138438	0.404810	-2.485138
H	4.681206	0.752725	1.317046
H	5.505695	1.648497	0.032281
H	4.694846	-1.301267	0.023500
H	6.245455	-0.600960	-0.509546

Fig2-TS-2c

C	-1.310532	0.576193	-1.016132
C	-0.706467	1.988829	-1.164501
C	0.620947	2.005712	-0.344527
C	-0.295526	0.676922	1.394461
H	-0.426345	2.108001	-2.218769
O	0.300917	1.922352	1.044744
O	0.434858	-0.370614	1.330232
Li	1.172526	-1.620693	0.202860
H	-1.147061	0.587553	0.421178
C	-2.817134	0.437017	-1.092435
C	-3.443802	0.691957	-2.322099
C	-3.597594	0.034359	-0.004971
C	-4.823649	0.550046	-2.458232
H	-2.842497	1.002551	-3.171894
C	-4.980119	-0.110118	-0.142291
H	-3.126659	-0.167576	0.951175
C	-5.597346	0.147335	-1.366209
H	-5.294955	0.754368	-3.416187
H	-5.572180	-0.424813	0.712936
H	-6.673208	0.035608	-1.471107
S	-1.051125	-1.967444	-1.577547
O	-0.063719	-2.783365	-0.711703

C	-0.498780	-2.362541	-3.337285
C	-0.884061	-3.830441	-3.558710
H	-0.387634	-4.476713	-2.828259
H	-1.967273	-3.979867	-3.476198
H	-0.575350	-4.143650	-4.563582
C	1.013694	-2.164087	-3.442982
H	1.536250	-2.829792	-2.750005
H	1.340105	-2.398612	-4.464136
H	1.290474	-1.133357	-3.206621
C	-1.266607	-1.433044	-4.280066
H	-1.057067	-1.712666	-5.319901
H	-0.960565	-0.393330	-4.133225
H	-2.349731	-1.500947	-4.123187
C	-1.196365	0.852640	2.598750
C	-1.644100	-0.299048	3.258184
C	-1.636811	2.107250	3.033638
C	-2.520696	-0.199459	4.337721
H	-1.298051	-1.263837	2.903579
C	-2.506332	2.206583	4.121430
H	-1.287575	2.997454	2.522999
C	-2.953604	1.055886	4.774203
H	-2.864937	-1.100201	4.839894
H	-2.836580	3.185899	4.458618
H	-3.635289	1.136217	5.616974
C	-1.657224	3.113753	-0.746953
H	-2.506404	3.188928	-1.431359
H	-2.053136	2.939987	0.257427
H	-1.129632	4.072669	-0.743611
N	-0.493091	-0.376419	-1.510271
C	1.465970	3.230709	-0.595010
C	1.555341	4.277657	0.327990
C	2.178262	3.324125	-1.798695
C	2.337375	5.400619	0.048030
H	1.021368	4.197064	1.268413
C	2.957106	4.445894	-2.080211
H	2.121573	2.506704	-2.515096
C	3.037979	5.490674	-1.155703
H	2.400062	6.206459	0.775086
H	3.505278	4.502469	-3.017253
H	3.646715	6.364956	-1.370967
H	1.185473	1.117561	-0.644522
O	2.885649	-0.853767	-0.508268
C	3.890577	-1.805535	-0.929167
C	3.499139	0.189016	0.286350
C	5.229080	-1.292664	-0.375584
H	3.621875	-2.777969	-0.503055
H	3.872712	-1.873863	-2.021943
C	4.784941	-0.434513	0.820057

H	3.708239	1.055765	-0.356148
H	2.774584	0.482747	1.048191
H	5.740215	-0.668204	-1.117863
H	5.903013	-2.108983	-0.098926
H	5.526201	0.313624	1.116811
H	4.563731	-1.068597	1.685935
O	2.039574	-2.962094	1.492955
C	1.323666	-4.202966	1.630840
C	2.205665	-2.415254	2.821058
C	0.282350	-3.902040	2.705884
H	0.902588	-4.443873	0.654637
H	2.019541	-4.995640	1.949086
C	1.068826	-3.007395	3.689800
H	3.197000	-2.698686	3.200383
H	2.142377	-1.330904	2.725742
H	-0.122230	-4.803979	3.175632
H	-0.541001	-3.347404	2.245136
H	1.480414	-3.600374	4.513535
H	0.444387	-2.221147	4.121017

Fig3-TS-2a-E

C	-1.572902	-0.482774	-1.163565
C	-0.322866	-1.208304	-1.711693
C	0.930877	-1.087467	-0.801107
C	-0.399547	-0.801911	1.144362
H	-0.564521	-2.275719	-1.714347
H	1.207766	-0.036077	-0.759140
O	0.629761	-1.585671	0.500280
O	-0.105803	0.447052	1.376163
Li	1.276441	1.512131	0.732828
H	-1.253154	-0.841323	0.276294
C	-2.865782	-1.269832	-1.189432
C	-3.103313	-2.312093	-2.094992
C	-3.874266	-0.927275	-0.275113
C	-4.324180	-2.990972	-2.090280
H	-2.348564	-2.589835	-2.823821
C	-5.083806	-1.614883	-0.259195
H	-3.683385	-0.123769	0.429313
C	-5.315226	-2.649563	-1.170681
H	-4.496473	-3.788838	-2.807965
H	-5.845768	-1.348362	0.468425
H	-6.260082	-3.186461	-1.160711
S	-0.880284	2.156519	-0.830101
O	0.651759	2.068930	-1.067123
C	-1.491282	3.233074	-2.269194
C	-1.160217	2.582982	-3.608547
H	-1.675638	1.625086	-3.716126
H	-1.483488	3.241639	-4.424563

H	-0.082697	2.420962	-3.706320
C	-0.742843	4.561401	-2.103244
H	-0.955302	5.026364	-1.132942
H	0.337769	4.417756	-2.191744
H	-1.066735	5.259935	-2.883972
C	-3.000580	3.421423	-2.084620
H	-3.234248	3.843009	-1.099410
H	-3.531473	2.471792	-2.184320
H	-3.374035	4.118913	-2.845148
C	2.117071	-1.860179	-1.327905
C	2.281801	-3.218987	-1.036767
C	3.083710	-1.210263	-2.104772
C	3.396891	-3.913007	-1.509531
H	1.546885	-3.715285	-0.411238
C	4.194415	-1.903937	-2.586829
H	2.963336	-0.152046	-2.322214
C	4.356874	-3.258321	-2.285486
H	3.520018	-4.965499	-1.266971
H	4.937100	-1.385415	-3.188109
H	5.226438	-3.798922	-2.649949
C	-0.972803	-1.622443	2.281875
C	-1.363028	-0.981717	3.460882
C	-1.186375	-2.999530	2.145522
C	-1.944882	-1.709850	4.499960
H	-1.203628	0.088326	3.543402
C	-1.767383	-3.727756	3.182502
H	-0.892706	-3.489804	1.222663
C	-2.147754	-3.084756	4.364279
H	-2.241656	-1.204120	5.415845
H	-1.926523	-4.797417	3.069744
H	-2.602707	-3.652591	5.172079
C	-0.025392	-0.745139	-3.145386
H	0.700222	-1.409736	-3.622451
H	-0.936776	-0.733812	-3.752030
H	0.387681	0.266126	-3.131855
N	-1.800221	0.817450	-1.134849
O	1.321524	3.065097	1.976548
C	0.632628	2.781716	3.210606
C	0.825795	4.334426	1.506546
C	-0.805699	3.217081	2.935084
H	0.737179	1.712249	3.398761
H	1.096551	3.358551	4.025387
C	-0.627777	4.458592	2.027597
H	1.464541	5.134618	1.904633
H	0.909125	4.321483	0.417994
H	-1.366237	3.434137	3.849567
H	-1.302958	2.411486	2.389125
H	-0.759102	5.391791	2.584780

H	-1.351190	4.451036	1.208056
O	3.041476	0.689504	0.782007
C	4.266192	1.237399	0.270729
C	3.378343	-0.308620	1.763240
C	5.334965	0.126187	0.415163
H	4.075896	1.532287	-0.764731
H	4.532198	2.133202	0.848845
C	4.603756	-1.008417	1.174061
H	3.609501	0.184496	2.720554
H	2.502940	-0.948675	1.873847
H	6.199350	0.496342	0.975608
H	5.689626	-0.220589	-0.558785
H	5.226721	-1.470627	1.945811
H	4.282741	-1.786506	0.477812

Fig4-TS-3a-E

C	1.431326	-0.806008	0.455495
C	2.032207	0.346574	1.288889
C	1.526811	1.755289	0.885978
C	0.864332	1.022053	-1.257910
H	3.118192	0.334067	1.206517
H	0.460565	1.829423	1.122551
O	1.691643	1.905554	-0.525204
O	-0.382073	1.039242	-0.995435
Li	-2.024880	0.286211	-0.775404
H	1.316698	-0.107933	-0.733066
C	2.412654	-1.857832	-0.039938
C	1.993658	-3.187975	-0.191062
C	3.736427	-1.538719	-0.382297
C	2.868746	-4.166275	-0.662567
H	0.972627	-3.437242	0.070815
C	4.612493	-2.516117	-0.852821
H	4.087747	-0.514873	-0.307674
C	4.183791	-3.837230	-0.994305
H	2.520528	-5.190835	-0.767657
H	5.631570	-2.241306	-1.112229
H	4.866893	-4.599480	-1.359550
O	-2.221145	-0.441986	0.979926
N	0.200292	-1.326219	0.717914
S	-0.857508	-0.514675	1.711941
C	-1.168579	-1.861343	2.993324
C	-2.215598	-1.284146	3.952762
H	-1.856912	-0.365848	4.433986
H	-2.436336	-2.013088	4.742271
H	-3.144177	-1.056358	3.421077
C	-1.688146	-3.117120	2.295690
H	-2.614764	-2.900445	1.757666
H	-0.951366	-3.497516	1.583417

H	-1.890978	-3.895464	3.041952
C	0.165512	-2.114179	3.700933
H	0.025297	-2.853136	4.499165
H	0.557114	-1.198366	4.161928
H	0.913156	-2.500226	3.001339
C	1.312295	0.972963	-2.740825
C	2.835086	0.832383	-2.877807
C	0.614013	-0.220083	-3.412437
C	0.852505	2.293682	-3.391606
H	3.357446	1.639272	-2.356329
H	3.181491	-0.122097	-2.471939
H	3.117566	0.865420	-3.937210
H	-0.473542	-0.133682	-3.341442
H	0.893393	-0.273599	-4.471556
H	0.911702	-1.162343	-2.936263
H	1.121884	2.307009	-4.454639
H	-0.232571	2.406208	-3.309261
H	1.326377	3.152414	-2.904036
C	2.243923	2.938744	1.579210
C	2.109657	2.793549	3.106568
C	3.730222	3.013564	1.184525
C	1.538737	4.236842	1.135795
H	1.061316	2.658562	3.401856
H	2.682504	1.941974	3.489898
H	2.485248	3.693942	3.606525
H	3.839162	3.079849	0.098002
H	4.196746	3.898563	1.633254
H	4.291397	2.138305	1.531459
H	2.006839	5.110058	1.605676
H	1.592359	4.355894	0.049998
H	0.479826	4.225901	1.422450
H	1.793158	0.164995	2.340132
O	-3.344933	1.804467	-0.802183
C	-3.645435	2.297435	0.517688
C	-2.848582	2.941502	-1.533835
C	-2.479072	3.237780	0.855704
H	-4.604309	2.836925	0.486387
H	-3.722289	1.429915	1.173203
C	-1.997093	3.746578	-0.531286
H	-2.283973	2.555909	-2.384133
H	-3.704411	3.525903	-1.902393
H	-1.684360	2.671015	1.348809
H	-2.786891	4.047176	1.524968
H	-0.938950	3.521725	-0.671666
H	-2.148043	4.823226	-0.656812
O	-2.624848	-1.047332	-2.085319
C	-4.034454	-1.324304	-1.992410
C	-1.893251	-2.293917	-1.906603

C	-4.126065	-2.461462	-0.978260
H	-4.523420	-0.395658	-1.686856
H	-4.417796	-1.627083	-2.978897
C	-2.908167	-3.322719	-1.364123
H	-1.471299	-2.590244	-2.873372
H	-1.083054	-2.090469	-1.201826
H	-5.073769	-3.006763	-1.029822
H	-3.996650	-2.052538	0.028154
H	-3.181184	-4.046322	-2.140718
H	-2.503896	-3.877646	-0.513444

Fig4-TS-3b

C	1.552611	0.456386	-0.479114
C	1.266337	1.859277	-1.014363
C	-0.085381	2.398051	-0.501106
C	0.132028	1.223120	1.571740
H	1.256465	1.812776	-2.107833
O	-0.009299	2.483892	0.918609
O	-0.812811	0.375029	1.428888
Li	-1.601774	-0.853950	0.301552
H	1.146404	0.768795	0.900008
C	3.013196	0.127541	-0.237996
C	3.968027	0.547260	-1.176212
C	3.443204	-0.639928	0.852305
C	5.312890	0.204819	-1.030590
H	3.656854	1.137654	-2.033081
C	4.787325	-0.979941	1.002581
H	2.717957	-0.970347	1.586193
C	5.728185	-0.561021	0.059804
H	6.035705	0.538731	-1.770447
H	5.098356	-1.569800	1.860617
H	6.775765	-0.824939	0.176639
S	0.833467	-2.072902	-0.481298
O	-0.603898	-2.489259	-0.106220
C	1.104912	-2.886448	-2.158855
C	1.106169	-4.393951	-1.876956
H	0.149942	-4.709897	-1.449066
H	1.907424	-4.672725	-1.181982
H	1.266440	-4.942666	-2.813202
C	-0.038848	-2.499133	-3.094019
H	-1.002146	-2.760022	-2.644979
H	0.064170	-3.038551	-4.043912
H	-0.028690	-1.425182	-3.295455
C	2.463262	-2.408969	-2.677468
H	2.698364	-2.926506	-3.615680
H	2.453959	-1.332765	-2.870764
H	3.268700	-2.618263	-1.963824
N	0.674754	-0.458123	-0.950387

H	-0.871354	1.683697	-0.778245
C	0.685029	1.491108	2.999885
C	0.878319	0.150686	3.722424
C	2.015020	2.258017	2.935616
C	-0.371089	2.326802	3.752999
H	-0.061366	-0.403567	3.777031
H	1.608090	-0.482189	3.206446
H	1.246794	0.319420	4.741491
H	1.890174	3.211189	2.414399
H	2.387751	2.461648	3.947038
H	2.780761	1.679495	2.405515
H	-0.052562	2.498601	4.788773
H	-0.517549	3.296699	3.268845
H	-1.333355	1.803574	3.768623
C	-0.485477	3.796904	-1.034733
C	-1.890582	4.124272	-0.491059
C	0.500875	4.880962	-0.562418
C	-0.536092	3.775786	-2.573975
H	-2.629056	3.398820	-0.853737
H	-1.899249	4.092836	0.601759
H	-2.210346	5.121278	-0.817408
H	1.508550	4.714792	-0.960086
H	0.171249	5.869386	-0.904650
H	0.564720	4.896183	0.529211
H	-0.989483	4.699983	-2.951190
H	0.462345	3.696403	-3.017661
H	-1.134957	2.934021	-2.943238
H	2.063205	2.538528	-0.699413
O	-2.843171	-0.177706	-1.073405
C	-4.013665	0.545127	-0.639110
C	-2.596129	0.055088	-2.475926
C	-4.783895	0.853746	-1.921872
H	-4.544055	-0.088987	0.073766
H	-3.696620	1.463147	-0.126376
C	-3.639158	1.088052	-2.921347
H	-1.560423	0.387057	-2.590951
H	-2.714786	-0.898050	-3.005118
H	-5.450427	1.715185	-1.817038
H	-5.385007	-0.012777	-2.222458
H	-3.246901	2.104798	-2.809023
H	-3.937244	0.955074	-3.965675
O	-3.075325	-1.691526	1.465582
C	-2.761222	-1.625715	2.866964
C	-3.269099	-3.085977	1.156312
C	-1.808767	-2.802579	3.088031
H	-2.318079	-0.645439	3.045272
H	-3.686322	-1.734574	3.455444
C	-2.350125	-3.880100	2.115537

H	-4.328387	-3.338194	1.309018
H	-3.012275	-3.217964	0.104089
H	-1.784567	-3.135646	4.130445
H	-0.799858	-2.504869	2.790685
H	-2.915115	-4.655797	2.643157
H	-1.534532	-4.353802	1.566736

Fig4-TS-3a-Z

C	-1.454733	0.668066	-0.626671
C	-0.979254	2.131266	-0.661046
C	0.306814	2.356594	0.150584
C	-0.352177	0.661796	1.707961
H	-1.775887	2.765846	-0.261592
H	1.086342	1.692292	-0.233330
O	0.015897	2.025073	1.508421
O	0.506274	-0.233315	1.375390
Li	1.397439	-0.716446	-0.276896
H	-1.302212	0.572642	0.842329
C	-2.936338	0.533966	-0.887879
C	-3.361672	0.230531	-2.189834
C	-3.903472	0.772315	0.095074
C	-4.721073	0.135754	-2.490698
H	-2.622306	0.065512	-2.967446
C	-5.262015	0.671431	-0.201966
H	-3.587373	1.010566	1.104363
C	-5.676880	0.347338	-1.495599
H	-5.031578	-0.104245	-3.504257
H	-5.996895	0.842005	0.580309
H	-6.735467	0.265424	-1.726628
O	0.771976	-2.165822	-1.720419
N	-0.548960	-0.201406	-1.088681
S	-0.709156	-1.735717	-1.762564
C	-1.510070	-3.019770	-0.574235
C	-1.583160	-4.279826	-1.449705
H	-2.247272	-4.138875	-2.310764
H	-1.980267	-5.109213	-0.850599
H	-0.591874	-4.565111	-1.815322
C	-0.587748	-3.230920	0.620958
H	0.360489	-3.666914	0.302082
H	-0.378048	-2.287058	1.128864
H	-1.066254	-3.920274	1.329108
C	-2.907266	-2.580584	-0.145272
H	-3.391654	-3.412781	0.382108
H	-3.540288	-2.307933	-0.995359
H	-2.867281	-1.732298	0.537928
C	-1.001540	0.558231	3.120675
C	-1.914354	1.760073	3.412804
C	-1.805514	-0.745263	3.223409

C	0.151223	0.532842	4.144911
H	-1.340803	2.688338	3.465269
H	-2.674932	1.891640	2.635583
H	-2.433806	1.616065	4.368406
H	-1.177769	-1.613850	3.009856
H	-2.215915	-0.859893	4.234021
H	-2.646808	-0.752531	2.520527
H	-0.244031	0.484592	5.167339
H	0.792092	-0.337711	3.975961
H	0.765697	1.434844	4.053931
C	0.875538	3.796804	0.126374
C	1.290661	4.159868	-1.311577
C	-0.145393	4.819302	0.656318
C	2.130573	3.821332	1.022045
H	1.985920	3.414780	-1.717218
H	0.430306	4.224941	-1.986849
H	1.793765	5.134077	-1.326957
H	-0.499851	4.528813	1.650036
H	0.311246	5.813737	0.729049
H	-1.015416	4.905088	-0.004782
H	2.593976	4.815413	1.009992
H	1.877696	3.564979	2.053973
H	2.874232	3.096007	0.668558
H	-0.826473	2.395628	-1.712867
O	2.576788	0.472772	-1.406620
C	3.979301	0.695212	-1.177670
C	2.280996	0.533857	-2.823882
C	4.479818	1.397861	-2.438263
H	4.078838	1.289866	-0.264493
H	4.477010	-0.269490	-1.019723
C	3.634186	0.711203	-3.523004
H	1.756833	-0.384103	-3.095728
H	1.616914	1.390363	-2.990485
H	5.558002	1.283966	-2.587004
H	4.249386	2.469265	-2.392639
H	4.064602	-0.265585	-3.773722
H	3.554236	1.293403	-4.445984
O	2.982854	-1.677533	0.579565
C	3.213937	-3.069417	0.283662
C	3.195612	-1.491395	1.987410
C	2.963998	-3.845197	1.603214
H	2.532546	-3.331674	-0.527593
H	4.249527	-3.194661	-0.062351
C	2.580747	-2.740152	2.613877
H	4.276285	-1.421083	2.192121
H	2.696325	-0.563659	2.267372
H	3.872834	-4.368130	1.919527
H	2.173754	-4.593441	1.496528

H	2.947786	-2.939235	3.625540
H	1.495927	-2.607828	2.652934

ig5-TS-4a

C	-0.303655	0.477207	1.509034
C	-1.756351	-0.023120	1.429142
C	-2.237404	-0.444879	0.007589
C	-0.605315	0.893551	-1.030363
H	-2.436002	0.754837	1.782428
H	-1.671904	-1.321489	-0.322175
O	-2.007460	0.622704	-0.898010
O	0.135581	-0.085020	-1.418980
Li	1.757880	-0.912886	-1.394591
H	-0.331566	1.083990	0.172758
C	-0.079508	1.797718	2.210407
C	1.023497	1.970139	3.058309
C	-0.944668	2.884094	2.004396
C	1.248242	3.192181	3.692062
H	1.696422	1.134935	3.211799
C	-0.716237	4.106182	2.631846
H	-1.782730	2.782822	1.322664
C	0.380459	4.265068	3.482517
H	2.104738	3.304881	4.351903
H	-1.391199	4.937854	2.448801
H	0.557749	5.217921	3.974066
O	1.836828	-2.163718	0.071180
C	-3.709868	-0.783699	0.038625
C	-4.111402	-2.037887	0.514869
C	-4.684800	0.140582	-0.349841
C	-5.464246	-2.363046	0.609231
H	-3.355865	-2.764577	0.808262
C	-6.039360	-0.186065	-0.261773
H	-4.373126	1.104836	-0.736700
C	-6.433780	-1.435417	0.220524
H	-5.761425	-3.341444	0.977969
H	-6.788520	0.537388	-0.573454
H	-7.488773	-1.687667	0.287833
C	-0.425243	2.256969	-1.666638
C	0.760482	2.530451	-2.358314
C	-1.388614	3.264594	-1.533666
C	0.980401	3.794816	-2.905730
H	1.504643	1.748117	-2.459919
C	-1.169398	4.526845	-2.084984
H	-2.315060	3.046836	-1.013522
C	0.017966	4.797691	-2.769279

H	1.904339	3.995889	-3.442402
H	-1.927136	5.299678	-1.982075
H	0.189953	5.782720	-3.195606
N	0.768239	-0.327827	1.528564
S	0.638444	-1.925767	1.014383
C	1.169546	-2.798707	2.596326
C	0.085975	-2.520184	3.642037
H	0.026947	-1.452171	3.874160
H	0.322553	-3.058141	4.567796
H	-0.900533	-2.861315	3.303811
C	1.230634	-4.287241	2.232062
H	0.259596	-4.656319	1.879584
H	1.973619	-4.464771	1.449096
H	1.510935	-4.871230	3.117132
C	2.536052	-2.282131	3.042465
H	2.870982	-2.847388	3.921090
H	2.486884	-1.223386	3.308010
H	3.274257	-2.408969	2.246022
H	-1.873180	-0.875987	2.105372
O	3.261995	0.361035	-1.365181
C	3.222818	1.264316	-0.235097
C	4.564872	-0.244514	-1.351408
C	4.107697	0.623511	0.855104
H	3.604039	2.241994	-0.558232
H	2.179296	1.365159	0.061475
C	4.827197	-0.541930	0.127855
H	4.520433	-1.126714	-1.995708
H	5.300016	0.463696	-1.763853
H	3.484914	0.249637	1.669090
H	4.815608	1.351961	1.262865
H	4.353056	-1.491416	0.388815
H	5.895119	-0.601877	0.359399
O	1.754240	-1.992433	-3.062629
C	0.864434	-1.420653	-4.047924
C	1.286992	-3.335944	-2.838119
C	-0.526429	-2.041953	-3.787294
H	0.885663	-0.338912	-3.909552
H	1.243909	-1.672742	-5.047955
C	-0.239011	-3.206054	-2.803381
H	1.622544	-3.977686	-3.667311
H	1.727596	-3.677762	-1.900475
H	-0.977179	-2.396460	-4.719739
H	-1.193016	-1.308847	-3.330416
H	-0.742014	-4.137711	-3.080709
H	-0.554324	-2.929919	-1.793282

Fig5-TS-4b

C	-0.131398	-1.030005	-1.199555
C	1.372959	-1.262971	-1.336497
C	2.126689	-0.574641	-0.171500
C	0.339873	-0.946650	1.333920
H	1.601327	-2.332982	-1.311959
H	1.831951	0.480021	-0.155586
O	1.719622	-1.185506	1.047824
O	-0.023616	0.257424	1.563287
Li	-0.536770	1.849983	0.757125
H	-0.136909	-1.257682	0.202005
C	-1.034024	-2.177180	-1.602173
C	-0.867750	-2.756873	-2.867690
C	-2.051008	-2.652188	-0.765426
C	-1.706386	-3.788379	-3.290464
H	-0.081675	-2.393738	-3.524508
C	-2.886429	-3.687080	-1.186368
H	-2.179747	-2.219645	0.221600
C	-2.718638	-4.256705	-2.449734
H	-1.566571	-4.227956	-4.274648
H	-3.666203	-4.049629	-0.522167
H	-3.369512	-5.063307	-2.776429
S	-2.102274	0.682536	-1.326456
O	-2.168697	1.833666	-0.299533
C	-2.257743	1.561689	-2.984125
C	-1.225499	2.685877	-3.056356
H	-0.210261	2.282515	-3.045081
H	-1.369724	3.257015	-3.982014
H	-1.339481	3.364343	-2.205169
C	-3.687014	2.117494	-3.009972
H	-4.432263	1.316958	-2.930624
H	-3.844551	2.821386	-2.187089
H	-3.858646	2.645367	-3.955989
C	-2.042284	0.507595	-4.072802
H	-2.731144	-0.338168	-3.962584
H	-1.019831	0.120107	-4.043960
H	-2.213869	0.958763	-5.057771
C	3.626826	-0.677467	-0.321410
C	4.402236	-1.479276	0.521282
C	4.260834	0.050378	-1.338840
C	5.786481	-1.552774	0.346723
H	3.915040	-2.029740	1.318240
C	5.641685	-0.022751	-1.513824
H	3.665378	0.682800	-1.994457

C	6.410661	-0.828013	-0.669196
H	6.378321	-2.177579	1.010831
H	6.117797	0.550205	-2.305466
H	7.487873	-0.885593	-0.800984
C	-0.200090	-2.065330	2.199009
C	0.357118	-3.349021	2.172243
C	-1.344726	-1.827711	2.967653
C	-0.215505	-4.377299	2.920510
H	1.236385	-3.530303	1.563701
C	-1.920378	-2.858180	3.710882
H	-1.773062	-0.831751	2.967176
C	-1.356363	-4.136116	3.690508
H	0.226531	-5.370236	2.899630
H	-2.808766	-2.663923	4.306736
H	-1.804088	-4.940103	4.269033
N	-0.465623	0.267280	-1.392224
C	-1.699971	2.170818	3.420462
C	-2.252019	3.857462	1.952944
C	-3.129230	1.831638	2.989071
H	-1.687858	2.765686	4.347603
H	-1.037598	1.310645	3.523894
C	-3.576223	3.103379	2.224418
H	-2.100475	4.101016	0.900070
H	-2.177543	4.775123	2.553647
H	-3.106975	0.978744	2.304800
H	-3.778425	1.586900	3.835709
H	-4.070907	2.839610	1.288180
H	-4.259359	3.716934	2.821030
O	-1.188743	2.964230	2.337176
O	1.012447	3.012853	0.350182
C	1.909514	3.094705	1.482980
C	1.739272	3.173273	-0.885618
C	3.310938	2.899701	0.906146
H	1.791527	4.079250	1.953793
H	1.612157	2.319239	2.192431
C	3.178878	3.529267	-0.490320
H	1.675591	2.230255	-1.440411
H	1.247508	3.951945	-1.477753
H	3.549729	1.835024	0.826157
H	4.083780	3.374672	1.518058
H	3.913588	3.140387	-1.201583
H	3.301978	4.617116	-0.432686
H	1.719241	-0.852520	-2.290676

Fig6-TS-5a

C	-0.067844	1.347672	1.690498
C	-1.467652	0.700879	1.729776
C	-1.696589	-0.201192	0.484299
C	-0.252421	1.081366	-0.885483
O	-1.578445	0.592089	-0.693591
O	0.692325	0.239869	-1.060961
Li	1.304955	-1.422695	-0.523720
H	-0.066636	1.566733	0.323172
O	1.051707	-1.875643	1.287448
C	-0.290198	2.316644	-1.754868
C	-1.479508	2.996704	-2.044167
C	0.920106	2.812108	-2.256146
C	-1.456764	4.149188	-2.831519
H	-2.416246	2.614087	-1.655243
C	0.942915	3.968114	-3.034463
H	1.836066	2.282102	-2.020320
C	-0.247114	4.640260	-3.326583
H	-2.386673	4.665028	-3.057081
H	1.889011	4.345365	-3.414353
H	-0.230919	5.541018	-3.934564
C	-2.462216	1.884306	1.845576
H	-2.930415	1.877465	2.835205
H	-3.270327	1.804314	1.113026
N	1.099618	0.733303	1.967874
S	1.095199	-0.857936	2.462332
C	2.883725	-0.928517	3.042851
C	3.013453	0.028229	4.232004
H	2.293156	-0.211039	5.023966
H	2.853362	1.062259	3.917360
H	4.020917	-0.058254	4.657790
C	3.815774	-0.536748	1.896392
H	3.602612	0.480679	1.557958
H	4.855058	-0.582445	2.246658
H	3.707700	-1.221131	1.050672
C	3.111218	-2.384280	3.468399
H	4.139269	-2.498932	3.832358
H	2.961493	-3.063656	2.624591
H	2.432196	-2.679617	4.277646
C	-0.208921	2.827180	2.130752
C	-1.631521	3.172505	1.646742
H	-1.600440	3.440991	0.585690
H	-2.052239	4.029244	2.185145
C	-0.130910	2.866028	3.672083
H	0.852523	2.529394	4.012464
H	-0.884745	2.220424	4.137783
H	-0.296296	3.889198	4.030645
C	0.878655	3.725289	1.537073
H	1.869600	3.358564	1.825068

H	0.770480	4.755033	1.898812
H	0.825827	3.742269	0.443506
C	-3.033955	-0.900874	0.497734
C	-3.218050	-1.978047	1.375879
C	-4.086503	-0.521330	-0.340719
C	-4.431512	-2.663277	1.415589
H	-2.397260	-2.286501	2.020138
C	-5.301503	-1.209842	-0.304453
H	-3.938314	0.298459	-1.035175
C	-5.478860	-2.281174	0.572167
H	-4.557289	-3.500311	2.097433
H	-6.109523	-0.909246	-0.966563
H	-6.423559	-2.817772	0.596774
H	-0.914476	-0.965040	0.479793
H	-1.525786	0.042763	2.605814
O	0.329990	-2.789257	-1.595267
C	-0.182077	-2.313182	-2.856433
C	-0.674517	-3.613103	-0.946040
C	-1.692725	-2.247598	-2.662490
H	0.278647	-1.340790	-3.042309
H	0.100692	-3.017916	-3.653635
C	-1.961832	-3.482205	-1.783334
H	-0.304550	-4.643885	-0.900763
H	-0.783057	-3.238759	0.074759
H	-2.242057	-2.269936	-3.608854
H	-1.947318	-1.327047	-2.132075
H	-2.104966	-4.372137	-2.407060
H	-2.847092	-3.360764	-1.153874
O	3.135139	-1.506376	-1.332402
C	3.414118	-2.407500	-2.418186
C	3.662607	-0.225640	-1.719525
C	3.345008	-1.557061	-3.710761
H	4.415985	-2.836259	-2.276199
H	2.670818	-3.204574	-2.366122
C	3.272750	-0.096304	-3.193704
H	3.204788	0.518857	-1.067842
H	4.754355	-0.225875	-1.578682
H	2.465385	-1.809021	-4.309709
H	4.228267	-1.726525	-4.334515
H	2.249690	0.283779	-3.250521
H	3.928352	0.584297	-3.745479

Fig6-TS-5b

C	-0.803121	-0.727363	-1.586721
C	-2.255810	-0.649918	-1.078391
C	-2.309873	-0.024998	0.344319
C	-0.401778	1.312097	0.012527
O	-1.798054	1.300701	0.299305

O	0.379723	0.764096	0.871167
Li	1.785877	-0.410931	1.028341
H	-0.411872	0.553843	-1.022411
O	2.483367	-1.283520	-0.511579
C	-0.010353	2.655980	-0.568513
C	1.348214	2.890286	-0.813277
C	-0.945443	3.637955	-0.911622
C	1.769770	4.087747	-1.387905
H	2.059364	2.112240	-0.560633
C	-0.521710	4.844776	-1.474143
H	-1.998312	3.451994	-0.732178
C	0.834006	5.073603	-1.715675
H	2.826796	4.250604	-1.584838
H	-1.254731	5.606711	-1.727080
H	1.159535	6.010125	-2.160868
C	-3.045910	0.136819	-2.148844
H	-3.721321	-0.537705	-2.685513
H	-3.666087	0.919293	-1.702769
N	-0.059687	-1.662460	-0.972256
S	1.511468	-1.931804	-1.525329
C	1.610633	-3.769765	-1.142186
C	1.229842	-4.031673	0.314470
H	0.177965	-3.796730	0.488794
H	1.395462	-5.092203	0.543284
H	1.837986	-3.431362	0.997157
C	0.660937	-4.478118	-2.112778
H	0.927159	-4.279006	-3.157997
H	-0.373136	-4.160406	-1.949346
H	0.719757	-5.561883	-1.954383
C	3.073089	-4.150559	-1.405908
H	3.207074	-5.225082	-1.232884
H	3.367624	-3.936111	-2.440524
H	3.741246	-3.603209	-0.734814
C	-0.791936	-0.264844	-3.077340
C	-1.986011	0.717708	-3.101768
H	-1.651699	1.695856	-2.738707
H	-2.360738	0.861867	-4.121775
C	-1.088088	-1.498021	-3.959652
H	-2.004724	-2.012900	-3.651553
H	-1.209440	-1.185381	-5.003890
H	-0.267584	-2.219965	-3.913149
C	0.483524	0.433626	-3.573458
H	1.316478	-0.261244	-3.702405
H	0.282633	0.897768	-4.547387
H	0.796548	1.225779	-2.886981
C	-3.705812	-0.014164	0.921903
C	-4.282704	-1.223996	1.330837
C	-4.448082	1.164573	1.045005

C	-5.578871	-1.258622	1.842341
H	-3.707501	-2.144494	1.250615
C	-5.746557	1.131739	1.558922
H	-3.995141	2.105153	0.751703
C	-6.317521	-0.077769	1.956277
H	-6.010741	-2.205137	2.157412
H	-6.311285	2.056099	1.651203
H	-7.327448	-0.101487	2.357153
H	-1.662318	-0.633050	0.981846
H	-2.622151	-1.676819	-0.976469
O	1.427306	-1.527705	2.693608
C	0.023815	-1.833272	2.814005
C	1.802010	-0.941556	3.949748
C	-0.588884	-0.699963	3.667573
H	-0.368674	-1.900487	1.799018
H	-0.081441	-2.810976	3.306682
C	0.646694	0.009281	4.283607
H	1.901233	-1.736729	4.705337
H	2.767671	-0.456129	3.803710
H	-1.258272	-1.104644	4.433094
H	-1.155402	-0.003975	3.047139
H	0.550481	0.186080	5.359414
H	0.806745	0.970570	3.787211
O	3.366942	0.613590	1.694944
C	4.640447	0.471988	1.044759
C	3.195610	1.992991	2.092986
C	4.822739	1.789638	0.294787
H	5.428193	0.326361	1.800336
H	4.574827	-0.408277	0.403279
C	4.219154	2.816905	1.276870
H	2.155935	2.248307	1.877214
H	3.371303	2.077204	3.173375
H	4.251495	1.755224	-0.638749
H	5.868214	1.998483	0.048053
H	3.741795	3.654925	0.762300
H	4.996810	3.223839	1.931924

Fig6-TS-5c

C	-0.809087	-0.681719	-1.638646
C	-2.089518	-0.864041	-0.816303
C	-1.838949	-0.525844	0.652441
C	-0.690590	1.505616	-0.151365
H	-2.823447	-0.117716	-1.140021
H	-0.913365	-1.008463	0.984732
O	-1.699387	0.907369	0.695234
O	0.518877	1.451000	0.276579
Li	1.772283	0.203001	0.698143
H	-0.754722	0.804482	-1.217721

S	1.791018	-1.358294	-1.760683
O	2.723336	-0.645729	-0.762041
C	2.101344	-3.188055	-1.366176
C	1.919408	-3.406874	0.136154
H	0.897949	-3.180349	0.449662
H	2.145924	-4.453234	0.376484
H	2.600172	-2.766929	0.703114
C	3.553888	-3.439391	-1.788907
H	3.700326	-3.260395	-2.860981
H	4.237524	-2.791246	-1.232776
H	3.819781	-4.483210	-1.582115
C	1.128018	-4.040804	-2.181823
H	1.278419	-3.912788	-3.259619
H	0.089190	-3.798498	-1.939181
H	1.293110	-5.100128	-1.950278
C	-2.976517	-0.928244	1.559641
C	-3.994365	-0.035540	1.911863
C	-3.036773	-2.247146	2.029129
C	-5.056346	-0.458163	2.714042
H	-3.933179	0.992150	1.570720
C	-4.100520	-2.671859	2.824710
H	-2.241003	-2.941894	1.767832
C	-5.116126	-1.776461	3.169651
H	-5.838311	0.246590	2.985996
H	-4.132843	-3.698058	3.182147
H	-5.943568	-2.102950	3.794284
C	-1.219620	2.844751	-0.640048
C	-2.587968	3.079498	-0.824054
C	-0.304021	3.844255	-0.984948
C	-3.031795	4.297141	-1.340626
H	-3.298648	2.307165	-0.550109
C	-0.747044	5.061519	-1.502876
H	0.752905	3.650388	-0.836890
C	-2.112879	5.292115	-1.683562
H	-4.096966	4.471312	-1.472532
H	-0.025897	5.832216	-1.764516
H	-2.459202	6.240401	-2.086705
C	-2.568779	-2.201662	-1.384453
H	-3.606270	-2.431115	-1.125313
H	-1.940259	-3.022433	-1.022178
N	0.258604	-1.231515	-1.066971
C	-1.302672	-0.801316	-3.109315
C	-2.364723	-1.968014	-2.917853
H	-2.022503	-2.880392	-3.417055
H	-3.310635	-1.679121	-3.389170
C	-1.974623	0.508492	-3.574699
H	-2.451027	0.353729	-4.550114
H	-2.740548	0.867983	-2.883422

H	-1.231854	1.306709	-3.679661
C	-0.260098	-1.180887	-4.166742
H	0.225846	-2.133116	-3.947346
H	-0.759375	-1.285625	-5.137992
H	0.514935	-0.415382	-4.269459
O	1.263689	-0.707022	2.414134
C	0.384107	0.058352	3.264379
C	2.359375	-1.128180	3.241896
C	1.332012	0.900100	4.122286
H	-0.273287	0.636421	2.615362
H	-0.214591	-0.634699	3.874657
C	2.579203	-0.006414	4.284018
H	2.097944	-2.081304	3.723473
H	3.218146	-1.291379	2.585860
H	0.883449	1.185968	5.078522
H	1.596887	1.811885	3.580081
H	2.656530	-0.422750	5.293475
H	3.493887	0.551025	4.071685
O	3.358776	1.298031	1.440428
C	4.647209	0.951031	0.901336
C	3.183396	2.698905	1.157502
C	4.806542	1.780158	-0.391484
H	5.420100	1.204245	1.642490
H	4.645883	-0.125998	0.727918
C	3.696152	2.859772	-0.277763
H	2.122394	2.920407	1.275478
H	3.778242	3.287890	1.873133
H	4.642696	1.148922	-1.266574
H	5.807131	2.219297	-0.457957
H	2.881839	2.635999	-0.971995
H	4.058756	3.872567	-0.479208

3. References for supporting information

1 Gaussian 16, Revision C.01, M. J. Frisch, G. W. Trucks, H. B. Schlegel, G. E. Scuseria, M. A. Robb, J. R. Cheeseman, G. Scalmani, V. Barone, G. A. Petersson, H. Nakatsuji, X. Li, M. Caricato, A. V. Marenich, J. Bloino, B. G. Janesko, R. Gomperts, B. Mennucci, H. P. Hratchian, J. V. Ortiz, A. F. Izmaylov, J. L. Sonnenberg, D. Williams-Young, F. Ding, F. Lipparini, F. Egidi, J. Goings, B. Peng, A. Petrone, T. Henderson, D. Ranasinghe, V. G. Zakrzewski, J. Gao, N. Rega, G. Zheng, W. Liang, M. Hada, M. Ehara, K. Toyota, R. Fukuda, J. Hasegawa, M. Ishida, T. Nakajima, Y. Honda, O. Kitao, H. Nakai, T. Vreven, K. Throssell, J. A., Jr. Montgomery, J. E. Peralta, F. Ogliaro, M. J. Bearpark, J. J. Heyd, E. N. Brothers, K. N. Kudin, V. N. Staroverov, T. A. Keith, R. Kobayashi, J. Normand, K. Raghavachari, A. P. Rendell, J. C. Burant, S. S. Iyengar, J. Tomasi, M. Cossi, J. M. Millam, M. Klene, C. Adamo, R. Cammi, J. W. Ochterski, R. L. Martin, K. Morokuma, O. Farkas, J. B. Foresman and D. J. Fox, Gaussian, Inc., Wallingford CT, 2016.

2 Spartan'16. Wavefunction, Inc. Irvine, CA.

3 Pracht, P.; Bohle, F.; Grimme, S. Automated exploration of the low-energy chemical space with fast quantum chemical methods. *Phys. Chem. Chem. Phys.* **2020**, *22*, 7169.

4 (a) Head-Gordon, M.; Pople, J. A.; Frisch, M. J. MP2 energy evaluation by direct methods. *Chem. Phys. Lett.* **1988**, *153*, 503. (b) Becke, A. D. Density-functional thermochemistry. III. The role of exact exchange. *J. Chem. Phys.* **1993**, *98*, 5648. (c) Lee, C.; Yang, W.; Parr, R. G. Development of the Colle-Salvetti correlation-energy formula into a functional of the electron density. *Phys. Rev. B* **1988**, *37*, 785. (d) Vosko, S. H.; Wilk, L.; Nusair, M. Accurate spin-dependent electron liquid correlation energies for local spin density calculations: a critical analysis. *Can. J. Phys.* **1980**, *58*, 1200. (e) Stephens, P. J.; Devlin, F. J.; Chabalowski, C. F.; Frisch, M. J. Ab Initio Calculation of Vibrational Absorption and Circular Dichroism Spectra Using Density Functional Force Fields. *J. Phys. Chem.* **1994**, *98*, 11623.

5 Grimme, S.; Antony, J.; Ehrlich, S.; Krieg, H. A consistent and accurate ab initio parametrization of density functional dispersion correction (DFT-D) for the 94 elements H-Pu. *J. Chem. Phys.* **2010**, *132*, 154104.

6 Zhao, Y.; Truhlar, D. G. The M06 suite of density functionals for main group thermochemistry, thermochemical kinetics, noncovalent interactions, excited states, and transition elements: two new functionals and systematic testing of four M06-class functionals and 12 other functionals. *Theor. Chem. Acc.* **2008**, *120*, 215.

7 R. S. Paton, J. Rodríguez-Guerra and J. I. Funes, GoodVibes: version 3.0.0; Zenodo, 2019; DOI: 10.5281/zenodo.3346166.

8 The PyMOL Molecular Graphics System, Version 2. Schrödinger, LLC.

9 GaussView, Version 6, Roy. Dennington, Todd A. Keith and John M. Millam, Semichem Inc., Shawnee Mission, KS, 2016.
